# Atomic Level Defect Structure Engineering for Unusually High Average Thermoelectric Figure of Merit in n‐Type PbSe Rivalling PbTe

**DOI:** 10.1002/advs.202203782

**Published:** 2022-10-26

**Authors:** Bangzhi Ge, Hyungseok Lee, Lulu Huang, Chongjian Zhou, Zhilei Wei, Bowen Cai, Sung‐Pyo Cho, Jing‐Feng Li, Guanjun Qiao, Xiaoying Qin, Zhongqi Shi, In Chung

**Affiliations:** ^1^ State Key Laboratory for Mechanical Behavior of Materials Xi'an Jiaotong University Xi'an 710049 China; ^2^ School of Chemical and Biological Engineering and Institute of Chemical Processes Seoul National University Seoul 08826 Republic of Korea; ^3^ Center for Correlated Electron Systems Institute for Basic Science (IBS) Seoul 08826 Republic of Korea; ^4^ Key Lab of Photovoltaic and Energy Conservation Materials Institute of Solid State Physics HFIPS Chinese Academy of Sciences Hefei 230031 China; ^5^ State Key Laboratory of New Ceramics and Fine Processing School of Materials Science and Engineering Tsinghua University Beijing 100190 China; ^6^ National Center for Inter‐University Research Facilities Seoul National University Seoul 08826 Republic of Korea; ^7^ School of Materials Science and Engineering Jiangsu University Zhenjiang 212013 China

**Keywords:** average thermoelectric figure of merit, nanoscale defect, PbSe, power factor, thermoelectric

## Abstract

Realizing high average thermoelectric figure of merit (ZT_ave_) and power factor (PF_ave_) has been the utmost task in thermoelectrics. Here the new strategy to independently improve constituent factors in ZT is reported, giving exceptionally high ZT_ave_ and PF_ave_ in n‐type PbSe. The nonstoichiometric, alloyed composition and resulting defect structures in new Pb_1+_ *_x_* Se_0.8_Te_0.2_ (*x* = 0–0.125) system is key to this achievement. First, incorporating excess Pb unusually increases carrier mobility (*µ*_H_) and concentration (*n*_H_) simultaneously in contrast to the general physics rule, thereby raising electrical conductivity (*σ*). Second, modifying charge scattering mechanism by the authors’ synthesis process boosts a magnitude of Seebeck coefficient (*S*) above theoretical expectations. Detouring the innate inverse proportionality between *n*_H_ and *µ*_H_; and *σ* and *S* enables independent control over them and change the typical trend of PF to temperature, giving remarkably high PF_ave_ ≈20 µW cm^−1^ K^−2^ from 300 to 823 K. The dual incorporation of Te and excess Pb generates unusual antisite Pb at the anionic site and displaced Pb from the ideal position, consequently suppressing lattice thermal conductivity. The best composition exhibits a ZT_ave_ of ≈1.2 from 400 to 823 K, one of the highest reported for all n‐type PbQ (Q = chalcogens) materials.

## Graphical abstract

The dual incorporation of Te and excess Pb generates unusual antisite Pb at the anionic site and displaces Pb from the ideal position, which extraordinarily enhances the power factor and depresses thermal conductivity, thereby boosting average ZT to 1.2 from 400 to 823 K, even comparable to the record‐high value for n‐type PbTe.

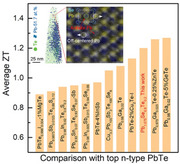

## 1 Introduction

Humanity faces a rapidly growing energy crisis. The price of crude oil increased by 65% in 2021, and gasoline costs the highest since 2014. The price of coal in the United States even surged up to 400% in 2021.^[^ ^1^ ^]^ However, the efficiency of energy use remains very low. Only one third from total energy input is being utilized practically and the rest is lost as waste heat.^[^ ^2^ ^]^ More seriously, electricity is generated mostly from fossil fuels such as coal and natural gas, causing critical environmental problems.^[^ ^3^ ^]^ Thermoelectrics emerges as a renewable energy technology that can address aforementioned energy and environmental problems simultaneously.^[^ ^4^ ^]^ Thermoelectric (TE) materials can generate electric energy spontaneously by Seebeck effect when subjected to a temperature gradient.^[^ ^5^ ^]^ Because TE power generators are electronic devices simply comprising n‐ and p‐type TE semiconductors, recovering waste heat does not emit any environmentally hazardous chemicals such as greenhouse gases.^[^ ^6^ ^]^ For broad applications of TE technology, improving power conversion performance has been the most important task in the TE community.^[^ ^6^ ^]^

The performance of TE materials is commonly estimated by a dimensionless figure of merit ZT = *σ S* ^2^ *T* / *κ*_tot_, where *σ* is the electrical conductivity, *S* is the Seebeck coefficient, the product *σ S* ^2^ is the power factor (PF), *T* is the absolute temperature, *κ*_tot_ is the total thermal conductivity that is a sum of the lattice (*κ*_lat_) and electrical thermal conductivity (*κ*_ele_).^[^ ^7^ ^]^ Recently, maximum ZT (ZT_max_) values have greatly increased by virtue of discovery of new TE systems^[^ ^8^ ^]^ and many innovative performance‐enhancing strategies^[^ ^5^, ^6^, ^7^, ^9^ ^]^ such as, to name a few, defect engineering,^[^ ^10^ ^]^ nanostructuring^[^ ^10^, ^11^ ^]^ and band engineering.^[^ ^11^, ^12^ ^]^

TE researches start to more focus on improving average ZT (ZT_ave_) and average PF (PF_ave_) for practical applications of this technology.^[^ ^13^ ^]^ First, ZT_ave_ is crucial in stable operation of TE power generators over a wide range of temperature given that many TE materials exhibit high ZT in the very narrow temperature range and ZT_ave_ reported greater than unity is still rare. Indeed, ZT_ave_ directly determines conversion efficiency (*η*) of TE power generators according to Equation (1):^[^ ^11^, ^13^ ^]^

(1) $$\eta =\frac{T_{h}-T_{c}}{T_{h}}\times \frac{\sqrt{1+{\mathrm{ZT}}_{\mathrm{ave}}}-1}{\sqrt{1+{\mathrm{ZT}}_{\mathrm{ave}}}+\frac{T_{c}}{T_{h}}}$$

Second, PF_ave_ of TE materials is directly proportional to output power density (*ω*) of their TE power generation as expressed in Equation (2):^[^ ^13a^ ^]^

(2) $$\omega =\frac{\left(T_{h}-T_{c}\right)}{4L}{\mathrm{PF}}_{\mathrm{ave}}$$

where *T*_h_ and *T*_c_ signify the hot and cold side temperatures and *L* is the length of the TE material leg in a device. Namely, ZT_max_ and *κ*_tot_ do not affect *η* and *ω*. If abundant heat is supplied at low price continuously, TE modules consisting of high PF_ave_ materials would be economically more viable because they can generate high *ω* under the given temperature difference.

PbTe has been a top‐performing TE material system in the intermediate temperature range (600–900 K).^[^ ^12^, ^14^ ^]^ Due to the high cost and scarcity in the Earth crust of Te element,^[^ ^15^ ^]^ PbSe has emerged as a promising alternative given its similar crystal, electronic and phonon structures to PbTe^[^ ^16^ ^]^ and nearly 50 times greater natural abundance of Se than Te.^[^ ^13b^ ^]^ Although PbSe‐based TE materials much underperform PbTe analogues, they have rapidly advanced recently mainly by applying similar effective strategies developed for the latter.^[^ ^11^, ^17^ ^]^

In PbQ (Q = Se, Te) TE systems, n‐type materials historically perform far inferior to their p‐type cousins.^[^ ^16a^ ^]^ The latter benefits from the convergence of adjacent bands by either chemical doping or alloying, thereby increasing *S* without losing *σ* and consequently improving PF and ZT.^[^ ^11a^ ^]^ The similar strategy cannot be applied to the former because of its single electronic conduction band near Fermi level inhibiting.^[^ ^16^, ^18^ ^]^ With the lack of effective strategies for higher PF, ZT of the former has been mostly increased by reducing *κ*_lat_.^[^ ^10^, ^17^, ^19^ ^]^ The resulting poorer PF of the former has led to its typically lower ZT than the latter. Very recently, few exceptional cases show that proper doping and alloying can either flatten a conduction band edge^[^ ^12^, ^19^ ^]^ or increase density of states (DOS) effective mass,^[^ ^13b^ ^]^ demonstrating that a new strategy can improve PF and ZT of n‐type PbSe TE system.

Here we report a new n‐type Pb_1+_ *_x_* Se_0.8_Te_0.2_ (*x* = 0–0.125) TE system involving the atomic‐to‐nanoscale‐to‐submicron‐scale multidimensional defect structures favorably controlling charge and thermal transport properties. As a result, the Pb_1.075_Se_0.8_Te_0.2_ phase exhibits a remarkably high ZT_ave_ of 1.20 from 400 to 823 K, one of the highest reported for all PbQ (Q = Te, Se, S) TE systems. PF_ave_ reaches ≈20 µW cm^−1^ K^−2^ from 300 to 823 K, which is an exceptional value for PbSe system.

Employing the unique defect structures driven by excess Pb addition and Te alloying, we chemically break the fundamental semiconductor physics rules restricting independent control of the core parameters in ZT, namely, intrinsic inverse proportionality between *µ*_H_ and *n*_H_; and *σ* and *S* at the specific concentration range.^[^ ^5a^ ^]^ Nonstoichiometric composition and high energy ball‐milling process jointly create a new way of increasing PF. First, the incorporation of nonstoichiometric excess Pb atoms favorably modulates charge carrier transport properties. It increases *µ*_H_ and *n*_H_ simultaneously, thereby raising *σ* substantially. It thus circumvents the otherwise unavoidable loss between them, and provides a unique way of increasing PF. Second, the title system synthesized by a high energy ball‐milling process exhibits a significantly increased magnitude of *S* (|*S*|) than the control sample with the same composition prepared by the conventional solid state synthesis method. This unusual enhancement is attributed to the dominant charge scattering mechanism changing from acoustic phonon scattering at in‐grain regions to ionized impurity scattering at grain boundaries. Overall, incorporating excess Pb with mechanical process boosts PF of the materials, giving one of the highest PF_ave_ for PbSe‐based TE systems. In comparison, recently published n‐type PbTe system, Cu_3.3_Pb_100_Sb_3_Te_100_Se_6_, shows a comparable PF_ave_ ≈19 µW cm^−1^ K^−2^ from 300 to 723 K.^[^ ^20^ ^]^

Excess Pb incorporation collaborates with alloyed Te to induce highly unusual defect structures. They form the antisite defect of Pb atoms at the anion site in rock‐salt structure. It resultantly displaces Pb atoms out of the ideal octahedral site. The induced defect structures, coupled with the reduced particle size by high‐energy ball milling (HEBM), depress *κ*_lat_ of the materials without sacrificing *σ*. All these effects jointly contribute to substantially high ZT_ave_ of our new system, rivaling the record‐high value to date for n‐type PbTe materials.^[^ ^21^ ^]^ Remarkably, the best composition exceeds ZT of unity above 473 K. Very importantly, we directly observed the aforementioned defect structures and identified unusual atomic positions employing an atomic resolution spherical aberration‐corrected scanning transmission electron microscope (Cs‐corrected STEM). This achievement helps understand defect formation mechanisms and elevate the predictability of defect structure designs for desirable properties of materials.

## 2 Results and Discussion

### 2.1 Design Principle and Crystal Structure

Undoped PbSe is an intrinsic p‐type semiconductor because of innate Pb vacancies (V_Pb_) at the cationic site in the PbSe crystal matrix.^[^ ^22^ ^]^ This fact makes it hard to stabilize n‐type PbSe materials.^[^ ^22^ ^]^ To design high performance n‐type PbSe TE system, we introduced excess Pb atoms up to 12.5 at% to fill V_Pb_.^[^ ^23^ ^]^ We further alloyed 20 at% Te, a larger congener in Group 16, because of the two reasons. First, the smaller bond dissociation energy of the Pb—Te (249.8 kJ mol^−1^) than the Pb—Se bond (302.0 kJ mol^−1^)^[^ ^24^ ^]^ may help better accommodate excess Pb in the crystal lattice.^[^ ^9a^ ^]^ Second, alloying heavier Te can reduce lattice thermal conductivity (*κ*_lat_) considerably.^[^ ^11^, ^25^ ^]^ The resulting new series Pb_1+_ *_x_* Se_0.8_Te_0.2_ (*x* = 0–0.125) was synthesized by HEBM of the starting reagents in an appropriate mixing ratio to reduce the grain size of crystallites for an additional phonon scattering mechanism, followed by spark plasma sintering (SPS)^[^ ^26^ ^]^ to fabricate dense pellet samples.

**Figure 1:**
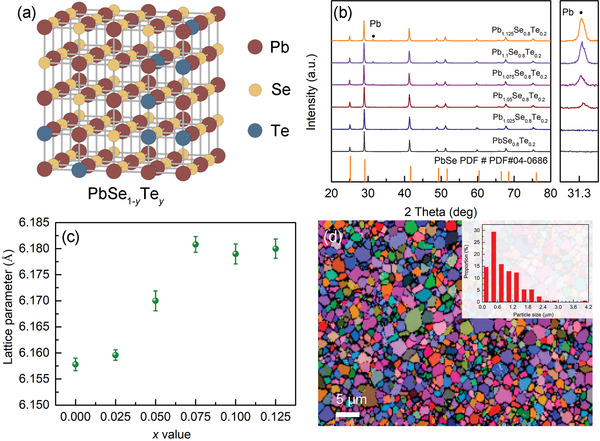
a) Local atomic structure of PbSe alloyed with Te atom (PbSe_1−_ *_y_* Te*_y_*). b) The powder X‐ray diffraction patterns and c) refined lattice parameters for the Pb_1+_ *_x_* Se_0.8_Te_0.2_ (*x* = 0–0.125) samples. d) Electron backscatter diffraction image for the SPS‐processed Pb_1.075_Se_0.8_Te_0.2_ sample. Inset shows a grain size distribution of the sample.

Because PbSe and PbTe are isostructural, their alloys form solid solutions of PbSe_1−_ *_y_* Te*_y_* (**Figure** **1**a). The powder X‐ray diffraction (PXRD) pattern of the PbSe_0.8_Te_0.2_ sample is fully indexed to the PbSe structure crystallizing in cubic rock‐salt structure with the *Fm* $\bar{3}$ *m* space group without any detectable impurity within the resolution of a laboratory X‐ray diffractometer (Figure 1b). Introducing excess Pb atom greater than 5 at% to the PbSe_0.8_Te_0.2_ matrix generates the Pb precipitate.

The refinement of the PXRD patterns for the Pb_1+_ *_x_* Se_0.8_Te_0.2_ (*x* = 0–0.125) samples shows that their lattice parameter increases with the larger *x* up to 0.075, and reaches a plateau afterward (Figure 1c). The *x* = 0.075 sample thus has a maximum amount of excess Pb in the crystal matrix, along with the evolving Pb precipitate. This observation indicates that a certain level of excess Pb is required to maximally insert Pb into the vacancy. The backscattered electron image on the surface of the SPS‐processed Pb_1.075_Se_0.8_Te_0.2_ sample shows that the size of embedded Pb precipitates is less than 1 µm (Figure S1, Supporting Information). Its electron backscatter diffraction (EBSD) image and the statistical size distribution show that the average grain size is ≈0.5 µm (Figure 1d and inset). It is much smaller than ≈25 µm in the control sample with the same composition, prepared by common melt synthesis and SPS process (Figure S2, Supporting Information). The obtained smaller grain size can effectively scatter low‐frequency phonon by grain boundary scattering, thereby suppressing *κ*_lat_ of the material at low temperatures as discussed later.^[^ ^10a^ ^]^

### 2.2 Direct Observation on Atomic‐Level Defect Structures

We investigated effects of the dual incorporation Te and excess Pb on local structures in the Pb_1.075_Se_0.8_Te_0.2_ sample using atom probe tomography (APT). It can give a 3D dispersal of participant elements in materials with the equal sensitivity quantitatively at a spatial resolution nearly down to the sub‐atomic level.^[^ ^13^, ^27^ ^]^

**Figure 2:**
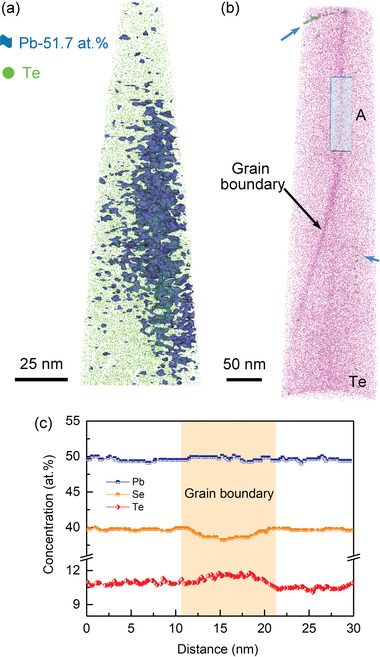
3D APT reconstruction and elemental analyses for the Pb_1.075_Se_0.8_Te_0.2_ sample. a) 3D spatial distribution of Pb and Te atoms. The blue isosurface of 51.7 at% Pb atom reveals Pb‐rich regions. b) 3D spatial distribution of Te atom demonstrates that the alloyed Te atoms are richer in grain boundaries. Green isosurface, indicated by the blue arrow, shows in‐grain Te‐rich regions, which are rarely observed throughout the specimens. c) 1D compositional profile showing the concentration of Pb, Se and Te atoms, taken across the grain boundary at area A in (b). The Te concentration increases as the Se concentration decreases across the grain boundary. Note that the composition outside a grain boundary is consistent with the nominal composition of the specimens.

**Figure** **2**a presents the 3D reconstruction of the needle‐formed specimen, showing the distribution of Pb and Te atoms. The blue isosurface of 51.7 at% Pb atom displays the presence of irregularly shaped and discrete nanoscale Pb‐rich regions randomly embedded in the matrix. They further aggregate into larger clusters with the size greater than 100 nm. This result shows how the Pb precipitate detected in our SEM observation (Figure S1, Supporting Information) forms. Te atom is generally more abundant at the grain boundary (dark pink area) than the in‐grain region (Figure 2b). The green isosurface of 20 at% Te reveals a short linear defect and tiny dots with even higher Te concentration as indicated by the blue arrow. However, they are so rarely found that we ignored them for major phonon scattering mechanism of the materials discussed later.

To resolve the compositional fluctuation quantitatively with a higher statistical accuracy, 1D compositional profiles were taken across the grain boundary in area A in Figure 2b (Figure 2c). The Te concentration slightly increases as the Se concentration decreases in a similar degree with the preservation of the Pb concentration. This observation indicates that Te atom, a larger Group 16 congener, resides more favorably at the grain boundaries possibly to reduce the total energy of the bulk matrix. Outside the grain boundary, the relative atomic ratio is close to the chemical composition of the specimen.

We further examined local structures in the Pb_1.075_Se_0.8_Te_0.2_ sample employing an atomic resolution spherical aberration‐corrected scanning transmission electron microscope (Cs‐corrected STEM) equipped with an energy‐dispersive spectroscopy (EDS) detector. All scanning TEM (STEM) images and electron diffraction (ED) patterns were taken parallel to the <110> zone axis of PbSe structure. Because the respective Pb and Se (Te) atoms are ordered linearly down this direction, they can be unambiguously discernable by their signal intensity and elemental maps in STEM and STEM‐EDS results.

**Figure 3:**
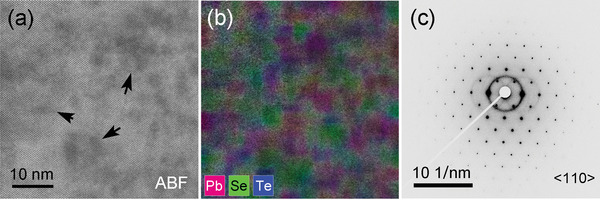
a) Low‐magnification ABF‐STEM image taken parallel to the <110> zone axis for the Pb_1.075_Se_0.8_Te_0.2_ sample. Nanoscale dark areas are embedded within the grain as indicated by the black arrow. b) Elemental map by STEM‐EDS scanned on (a), directly confirming the dark areas in (a) are Pb‐rich. It is overlaid with EDS signals directly arising from Pb (magenta), Se (green), and Te (blue) atoms, respectively. c) ED pattern taken at the same area in (a) along the <110> zone axis showing that this area is seemingly single phase.

The low‐magnification annular bright field (ABF) image presents that dark nanoscale regions are irregularly distributed in the surrounding matrix, indicated by the black arrow (**Figure** **3**a). The elemental map scanned on the corresponding area by STEM‐EDS reveals that such dark regions are abundant in the Pb atom (Figure 3b), consistent with our APT observation (Figure 2a). The experimental ED pattern (Figure 3c) taken at the same area shows a single set of the pattern corresponding to the rock‐salt structure down to the <110> zone axis, indicating that this area is apparently single phase without secondary impurity.

**Figure 4:**
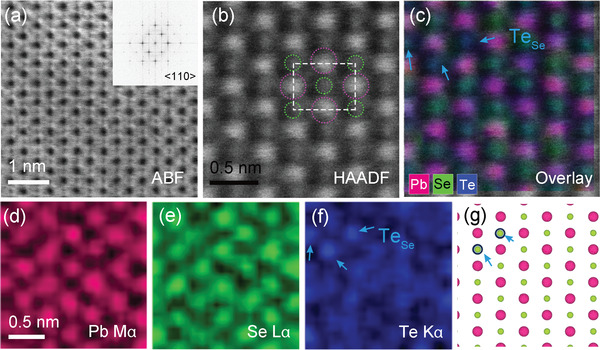
Atomic resolution local structure and direct elemental maps taken at the in‐grain, surrounding matrix down the <110> zone axis for the Pb_1.075_Se_0.8_Te_0.2_ sample. a) Medium‐magnification ABF‐STEM image. Inset: the corresponding FFT image in (a) showing single phase. b) HAADF‐STEM image differentiating bigger and brighter spheres from smaller and fainter ones. The Pb and Se (Te) atoms are tentatively assigned according to their signal intensity and marked by the magenta and green broken circles, respectively. The unit cell is given by the white broken line. c) Atomic resolution elemental map by STEM‐EDS scanned on (b). It is the overlapped EDS signals directly recorded from the d) Pb, e) Se, and f) Te atoms, respectively. The atomic sites for the constituent atoms are unambiguously identified directly. The Te atom replacing Se atoms (Te_Se_) is directly observed in (c) and (f) as indicated by the blue arrow, which cannot be distinguished in HAADF‐STEM images. g) Schematic illustration of Pb_1.075_Se_0.8_Te_0.2_ matrix structure viewed down the <110> zone axis based on the direct observation. The magenta, green and blue spheres represent Pb, Se and Te atoms, respectively.

The ABF‐STEM and corresponding fast Fourier transform (FFT) images on the nanostructure‐free area show an ideal rock‐salt crystal structure along the <110> zone axis without any discernible distortions and secondary phase (**Figure** **4**a and inset). The high‐magnification high‐angle annular dark‐field (HAADF)‐STEM image shows a periodic arrangement of brighter and bigger spheres and fainter and smaller ones (Figure 4b). The former and the latter can be assigned to the heavier ^82^Pb and the lighter ^34^Se or ^52^Te atoms, respectively, because the signal intensity in HAADF image is approximately proportional to the square of the atomic number.^[^ ^13b^ ^]^ They are marked by the broken circles (^82^Pb, magenta; ^34^Se, green) in the unit cell (white broken line). Their identification is clearly determined in the atomic resolution elemental map scanned on the same area by STEM‐EDS. It is the overlaid image of respective EDS data from the constituent atoms of ^82^Pb (magenta), ^34^Se (green), and ^52^Te (blue) (Figure 4d–f). It confirms their atomic positions definitely. It also shows the random disorder of ^34^Se and ^52^Te atoms at the same crystallographic sites marked by the blue arrow in Figures 4c and 4f, which cannot be resolved by HAADF‐STEM images. A schematic illustration of the observed structure is given in Figure 4g.

**Figure 5:**
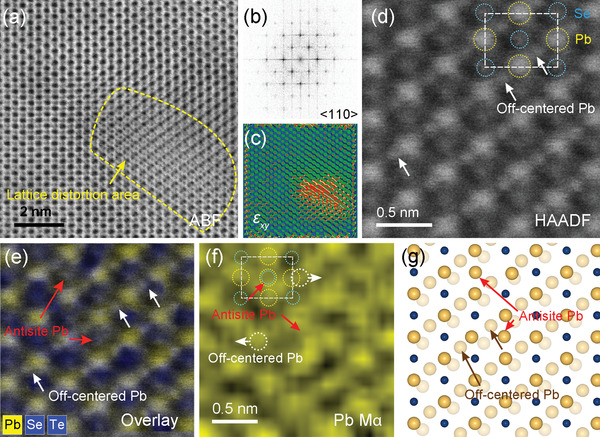
Atomic resolution local structure and direct elemental maps focusing on the nanoscale precipitate within the grain recorded down the <110> zone axis for the Pb_1.075_Se_0.8_Te_0.2_ sample. a) Medium‐magnification ABF‐STEM image displaying the presence of severe lattice distortion around the nanoscale precipitate enclosed by the orange dashed line. b) FFT image indicating seemingly single phase and c) shear strain (*ε*_xy_) map image generated by GPA presenting elastic strains accumulated inside the nanoscale precipitate, taken at the entire area in (a). d) High‐magnification HAADF‐STEM image focusing on the nanoscale precipitate. Pb and Se (Te) atoms are tentatively assigned according to their signal intensity and marked by the yellow and blue broken circles, respectively. The unit cell is drawn with the white broken line. The off‐centered atom displaced from the ideal octahedral site is marked by the white arrow, which is confirmed later as the Pb atom by STEM‐EDS in (e). e) Atomic resolution elemental map by STEM‐EDS scanned on the entire area in (d). It is the overlaid EDS signals directly collected from Pb (yellow) and Se or Te (blue) atoms, respectively. Off‐centered Pb and antisite Pb are directly observed. f) The elemental map of the Pb atom showing its location more clearly. The red arrows in (e) and (f) indicate the antisite Pb at the anionic sublattice. The white broken circle in (f) points out the off‐centered Pb due to electrostatic repulsion. g) The schematic illustration of local structure in the nanoscale precipitate viewed down the <110> zone axis. Yellow and blue spheres represent Pb and Se (Te) atoms, respectively.

The medium‐magnification ABF‐STEM image focusing on the Pb‐rich nanostructure (≈5 nm), outlined by the orange dashed line, reveals the presence of highly distorted the atomic arrays (**Figure** **5**a). The corresponding fast Fourier transform (FFT) image taken at the full area in Figure 5a, including the matrix and nanostructure, is matched to a single set of the diffraction spots along the <110> zone axis of the rock‐salt structure (Figure 5b and Figure S3, Supporting Information), which is consistent with our ED result in Figure 3c. It indicates that the nanostructure and the surrounding matrix form a highly coherent interface, and charge transport across them would not be damaged significantly. The corresponding shear strain (*ε*_xy_) map image is obtained by geometric phase analysis (GPA), which is a semiquantitative process from high‐quality TEM images and gives the distribution of strain fields (Figure 5c). This GPA result shows that elastic strains are accumulated inside the nanostructure and are almost negligible in the surrounding matrix. Accordingly, the Pb‐rich nanostructure induces elastic distortions of the crystal lattice, thereby significantly interrupting phonon transport.

The magnified HAADF‐STEM image (Figure 5d) and the elemental map on the Pb‐rich nanostructure by STEM‐EDS (Figure 5e) clearly reveal its strikingly unusual structural features. As discussed above, the bigger and brighter spheres can be assigned to the Pb atoms (marked by the yellow dotted circle), and thus the unit cell (white dotted line) can be defined with the smaller and fainter spheres (blue dotted circle) (Figure 5d). Then, many weak satellite signals near the Pb atoms, indicated by the white arrow, can be clearly differentiated. Their location does not correspond to the regular crystallographic site in the PbSe structure.

The elemental map clearly reveals those satellites as the Pb atom. Namely, such Pb atoms are displaced from their ideal crystallographic site along the <110> zone axis as indicated by the white arrow in Figures 5e and 5f. It also clarifies what makes Pb atoms off‐centered. To clearly distinguish the cations (Pb) and anions (Se and Te), the EDS signals of the former and the latter are displayed in yellow and blue color, respectively (Figure 5e). It strikingly reveals that several Pb atoms occupy the Se site as marked by the red arrow in Figures 5e and 5f. This antisite Pb (Pb_anion_) atom induces the electrostatic repulsion with the Pb atom at the regular crystallographic site, pushing the latter to be displaced. Namely, the introduction of the excess Pb atoms causes substantial lattice distortion in the Pb‐rich nanostructure. Figure 5g depicts a schematic local structure based on the HAADF‐STEM and STEM‐EDS observations in Figure 5d–f.

### 2.3 Defect Formation Mechanism

To better understand formation mechanism of the unusual defect structures revealed by our STEM observation, we calculated defect formation energy within the DFT regime. We generated the “Pb_32_Se_24_Te_8_” (PbSe_0.75_Te_0.25_) supercell for the alloyed crystal matrix. We considered all the possible defects in the supercell matrix: the vacancy at the Se (V_Se_), Te (V_Te_) and Pb sites (V_Pb_), interstitial Pb atom (Pb_i_), Pb replacing Se (Pb_Se_) and Te (Pb_Te_) atoms, and Te replacing Se (Te_Se_) atom. Figures S4a and S4b, Supporting Information, show the defect formation energy calculated as a function of Fermi energy for the Pb_32_Se_24_Te_8_ supercell matrix under both the Pb‐rich and Pb‐poor conditions, respectively. The result strongly supports the validity of our design principle for the Pb‐rich Pb_1+_ *_x_* Se_0.8_Te_0.2_ system. The Te_Se_ is very favorably generated thermodynamically, which is consistent with the previous experimental report that PbSe and PbTe readily form solid solutions throughout their entire composition range.^[^ ^25^ ^]^ V_Te_ becomes relatively favorable under the Pb‐rich condition as reported previously.^[^ ^28^ ^]^ It is more stable than V_Se_ under both the Pb‐rich and Pb‐poor conditions.

Note that a combined defect (Te_Se_ + Pb_Te_) is thermodynamically more favorable than a (Te_Se_ + Pb_Se_) couple as well as a single defect of either Pb_Se_ or Pb_Te_ under the Pb‐rich condition. Namely, the Pb_Te_ is more stable than the Pb_Se_ in the PbTe_0.25_Se_0.75_ crystal matrix if excess Pb is introduced. This result is consistent with the fact that Te is less electronegative than Se. Accordingly, the Pb_anion_ antisite defects observed in our HAADF‐STEM and STEM‐EDS results can be reasonably assigned to the Pb_Te_.

### 2.4 High Temperature Structure and Stability

We collected the in situ temperature‐dependent PXRD patterns from 300 to 698 K for the Pb_1.075_Se_0.8_Te_0.2_ sample to investigate its phase stability at the elevated temperatures (Figure S5a, Supporting Information). They involve the main rock‐salt phase and secondary Pb precipitate, and do not show chemical degradation upon heating. The cell dimension of the former increases almost linearly with the rising temperature (Figure S5b, Supporting Information), consistent with the behavior of PbQ (Q = Se and Te). These observations indicate that excess Pb atoms neither dissolve into the interstitial voids nor react with the matrix at high temperatures, validating excellent thermal stability of the material. Accordingly, excess Pb in the crystal matrix is expected to affect charge and thermal transport properties over the entire range of temperature.

The thermogravimetric analysis (TGA) curve for the Pb_1.075_Se_0.8_Te_0.2_ sample under an Ar flow at a rate of 10 K min^−1^ shows no thermal evaporation up to 850 K (Figure S5c, Supporting Information). Its differential scanning calorimetry (DSC) result presents the exothermic peak at ≈600 K on heating, which is slightly lower than that observed for the pure Pb reference specimen (Figure S5d, Supporting Information). The in situ temperature‐variant PXRD pattern shows the presence of secondary Pb phase above its melting point of ≈600 K (Figure S5a, Supporting Information), indicating that it partially remains as solid phase. Accordingly, the observed thermal event can be unambiguously ascribed to the partial melting of excess Pb embedded in the crystal matrix. Excess Pb in the crystal matrix reliably affect charge and thermal transport properties over the entire temperature range, which is confirmed by the measurement of thermoelectric properties on the round trip heating cycles as shown later.

### 2.5 Charge Transport Properties

**Figure 6:**
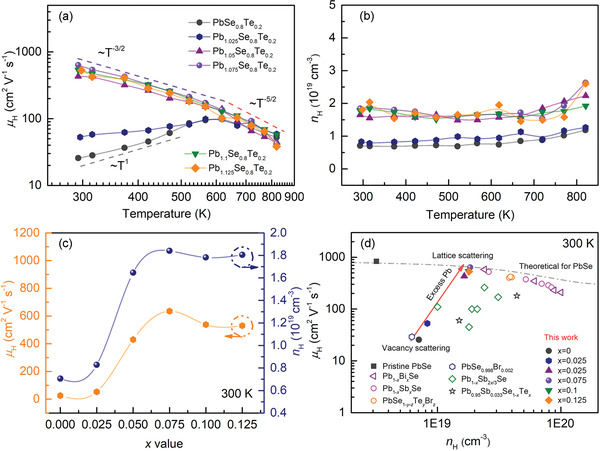
Charge transport properties of the Pb_1+_ *_x_* Se_0.8_Te_0.2_ samples (*x* = 0–0.125). a) Hall carrier mobility (*µ*_H_) and (b) concentration (*n*_H_) with respect to temperature. Purple, gray, and red dashed lines in (a) indicate the different power series of ≈*T* ^−3/2^, ≈*T* ^1^ and ≈*T* ^−5/2^, respectively, showing their distinct scattering mechanisms. c) The experimental *n*_H_ and *µ*_H_ with respect to the excess Pb content (*x*) at 300 K. d) The *µ*_H_ (orange) as a function of *n*_H_ (blue) of the title samples in comparison with the reported values of representative n‐type PbSe systems.^[^ ^10^, ^17^, ^22^, ^25^, ^30^ ^]^ The dashed curve is the theoretical *µ*_H_ for defect‐free, pristine PbSe from the previous report.^[^ ^17e^ ^]^

We conducted the temperature‐dependent Hall effect measurement for the Pb_1+_ *_x_* Se_0.8_Te_0.2_ (*x* = 0–0.125) samples to understand the effect of excess Pb on their charge transport properties (**Figure** **6**). Note that Pb precipitate was observed in the samples with *x* ≥ 0.05 according to our PXRD results (Figure 1b), and Pb‐rich areas contain the off‐centered and antisite Pb atoms in the crystal lattice (Figure 5). Such samples show a totally different trend in temperature‐dependent Hall carrier mobility (*µ*_H_) from those with *x* = 0 and 0.025 (Figure 6a). The former and latter exhibit *µ*_H_ following a different power series of ∼*T* ^−3/2^ (purple dashed line) and ∼*T* ^1^ (grey dashed line) from 300 to 623 K, indicating their distinct electron–phonon scattering mechanism, namely, scattering by vacancy and lattice,^[^ ^12^, ^17^ ^]^ respectively, according to Boltzmann transport theory.^[^ ^22^, ^29^ ^]^ This observation indicates that the sample with *x* = 0 and 0.025 contains intrinsic vacancy of the V_Te_ in the crystal matrix. It is consistent with our DFT calculation results (Figure S4, Supporting Information) and the previous report on PbSe_0.998_Br_0.002_ involving vacancy at the cationic site.^[^ ^22a^ ^]^ Adding more excess Pb atoms completely fills the V_Te_, and the induced Pb_Te_ consequently serves as one of the major defects in the samples with *x* ≥ 0.05. Namely, introducing sufficiently excess Pb atoms to the PbSe_0.8_Te_0.2_ lattice effectively changes charge scattering mechanism, thereby dramatically increasing *µ*_H_. Afterward, the *µ*_H_ of all samples trends ∼*T* ^−5/2^ (red dashed line) due to the electron–electron scattering mechanism at high temperatures,^[^ ^12^, ^17^ ^]^ as similarly reported in n‐type PbSe_0.998_Br_0.002_‐2%Cu_2_Se^[^ ^12a^ ^]^ and Pb_1−_ *_x_* Sb*_x_* Se^[^ ^17e^ ^]^ materials.

Very importantly, the samples with *x* ≥ 0.05 exhibit approximately twofold greater *n*_H_ than those with *x* = 0 and 0.025 despite the much higher *µ*_H_ in the full temperature range of measurements (Figure 6b). This observation shows two important features of these materials. First, in fact, the simultaneous increase in *µ*_H_ and *n*_H_ is in striking contrast to the general semiconductor physics in that they are inversely proportional each other because higher *n*_H_ brings lower *µ*_H_ inevitably because of greater electron–electron and electron‐defect scatterings. However, this has been a sought‐after goal in thermoelectrics because it can break the intrinsic inverse relationship between electrical conductivity and Seebeck coefficient, ultimately lead to higher ZT. Second, the increased *n*_H_ in the samples with *x* ≥ 0.05 confirms that the Pb_Te_, induced by a certain level of excess Pb atoms, serves as an electron donor to increase *n*_H_.

The *n*_H_ for all the Pb_1+_ *_x_* Se_0.8_Te_0.2_ (*x* = 0–0.125) samples is almost constant until ≈723 K, slightly increasing afterward because of bipolar effect.^[^ ^10b^ ^]^ This behavior indicates that excess Pb atoms do not dissolve into the crystal matrix on heating, consistent with the results of our thermal analysis and in situ temperature‐dependent PXRD (Figure S5, Supporting Information).

Figure 6c clearly shows that excess Pb concentration (*x*) simultaneously increases the *µ*_H_ and *n*_H_ values at 300 K. For example, the former and the latter rise from ≈25.64 cm^2^ V^−1^ s^−1^ and ≈0.71 × 10^19^ cm^−3^ for the *x* = 0 sample to ≈635.20 cm^2^ V^−1^ s^−1^ and ≈1.84 × 10^19^ cm^−3^ for the *x* = 0.075 sample. The following defect formation processes given by Equations (3) and (4) can show how *n*_H_ and *µ*_H_ increase simultaneously in the Pb_1+_ *_x_* Se_0.8_Te_0.2_ system (*x* ≥ 0.05):

(3) $${\mathrm{Te}}_{\mathrm{Te}}\underset{{\mathrm{PbSe}}_{1-y}{\mathrm{Te}}_{y}}{\to }\delta \mathrm{Te}+\delta V_{\mathrm{Te}}^{\cdot \cdot }+2\delta e$$

(4) $$\begin{array}{ccc} & & 2\delta \mathrm{Pb}+\delta {\mathrm{Te}}_{\mathrm{Te}}+{\mathrm{PbSe}}_{0.8}{\mathrm{Te}}_{0.2}\underset{\mathrm{Pb}-\mathrm{rich}}{\to }\left(1+\delta \right){\mathrm{PbSe}}_{0.8/\left(1+\delta \right)}{\mathrm{Te}}_{\left(0.2+\delta \right)/\left(1+\delta \right)}\\ & & \;+\;\delta {\mathrm{Pb}}_{\mathrm{Te}}^{\cdot \cdot \cdot \cdot }+4\delta e \end{array}$$

where Te_Te_ is the Te atom at the original position, $V_{\mathrm{Te}}^{\cdot \cdot }$ is the Te leaving its original position to form a Te vacancy with two negative charges, *e* is an electron with a negative charge, ${\mathrm{Pb}}_{\mathrm{Te}}^{\cdot \cdot \cdot \cdot }$ is the Pb_Te_ antisite defect providing four negative charges, and *δ* (0 < *δ* < *x*) is the coefficient for Te vacancy and Pb_Te_ antisite defect. “PbSe_0.8/(1+*δ*)_Te_(0.2+*δ*)/(1+*δ*)_” represents Te‐rich phase in the PbSe_0.8_Te_0.2_ surrounding matrix, which is observed in our experimental APT results (Figure 2b). Note that the excess Pb atoms remove the intrinsic V_Te_ and induce antisite defect Pb_Te_ according to our DFT calculations. This changes a major attribute to charge scattering mechanism from vacancy to lattice, consequently raising *µ*_H_.

To better understand the effect of the Pb_Te_ antisite defect on charge transport properties, we compare the experimental *µ*_H_ as a function of *n*_H_ at 300 K for our Pb_1+_ *_x_* Se_0.8_Te_0.2_ (*x* = 0–0.125) samples and representative n‐type PbSe systems from the previous reports^[^ ^10^, ^17^, ^25^, ^30^ ^]^ (Figure 6d). The theoretical *µ*_H_ for defect‐free, pristine PbSe from the previous report^[^ ^17e^ ^]^ is given as a reference for a lattice scattering mechanism (dashed line). Note that the *µ*_H_ increases with the greater *n*_H_ unusually, and fits on the theoretical curve at a given *n*_H_ with *x* = 0.075, representing its gradual change in scattering mechanism with the increasing excess Pb concentration. The *µ*_H_ of Pb_1−_ *_x_* Sb*_x_* Se involving point defect and dense nanostructures is slightly lower than the theoretical expectation. In contrast, PbSe_0.998_Br_0.002_ with intrinsic vacancy as well as Pb_1−_ *_x_* Sb_2*x*/3_Se^[^ ^30^ ^]^ and Pb_0.95_Sb_0.0033_Se_1−_ *_x_* Te*_x_* ^[^ ^10b^ ^]^ with artificially introduced high concentration vacancy are located far below the theoretical curve due to the serious damage in *µ*_H_ by vacancy and induced dislocation.^[^ ^10b^ ^]^

**Figure 7:**
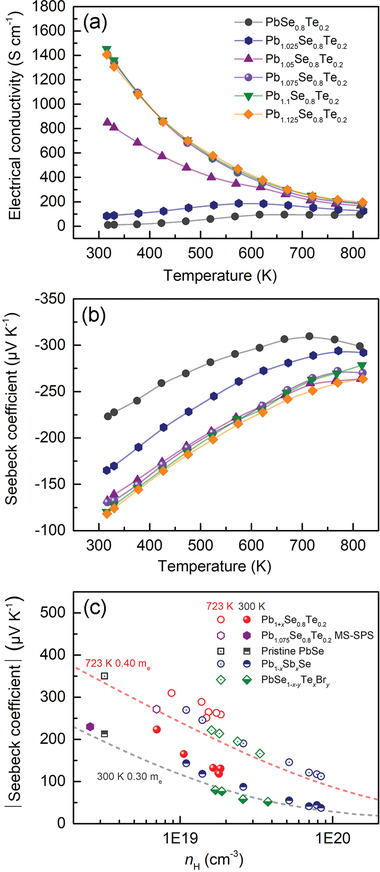
Temperature‐dependent a) electrical conductivity (*σ*) and b) Seebeck coefficient (*S*) for the Pb_1+_ *_x_* Se_0.8_Te_0.2_ (*x* = 0–0.125) samples. c) Theoretical Pisarenko lines assuming acoustic phonon scattering. Gray and red dashed lines are calculated for pristine PbSe based on SPB model with density of states effective mass (*m*_0_) of 0.30 *m*_e_ at 300 K and 0.40 *m*_e_ at 723 K. Experimental |*S*| values of the title Pb_1+_ *_x_* Se_0.8_Te_0.2_ (*x* = 0–0.125) samples (red circles) reside far above the theoretical Pisarenko lines for pristine PbSe (gray and red dashed lines) at given *n*_H_ at both 300 and 723 K. In contrast, the experimental data for pristine PbSe, Pb_1−_ *_x_* Sb*_x_* Se,^[^ ^17e^ ^]^ and PbSe_1−_ *_x_*_−_ *_y_* Te*_x_* Br*_y_* ^[^ ^25^ ^]^ from the previous reports and the control Pb_1.075_Se_0.8_Te_0.2_ sample prepared by traditional melt‐synthesis followed by SPS process (the black square, navy circle, green diamond, and purple hexagon, respectively) match well with the theoretical Pisarenko lines for pristine PbSe.

The concurrent increase in the *µ*_H_ and *n*_H_ by the addition of excess Pb is directly reflected in the dramatically boosted temperature‐dependent electrical conductivity (*σ*) for the Pb_1+_ *_x_* Se_0.8_Te_0.2_ samples (*x* ≥ 0.05) given the relation *σ* = *e* *µ*_H_ *n*_H_ (**Figure** **7**a). This evidently shows the effectiveness of our performance‐enhancing strategy for charge transport properties. For example, the *σ* value increases from 9.77 S cm^−1^ for the *x* = 0 sample to 1446.08 S cm^−1^ for *x* = 0.075 sample at 300 K. In contrast, Zn_0.0025_PbSe_0.998_Br_0.002_,^[^ ^22a^ ^]^ Pb_0.96_Sb_0.027_Se,^[^ ^30^ ^]^ Pb_0.9955_Sb_0.0045_Se‐12%GeSe^[^ ^17b^ ^]^ and Pb_0.99_Sb_0.01_Se‐3%CdSe^[^ ^19^ ^]^ from the previous reports present similar *n*_H_ of ≈2 × 10^19^ cm^−3^, but show much lower *σ* of ≈28, 310, 960 and 1005 S cm^−1^ at 300 K, respectively, due to the lower *µ*_H_ as commonly expected.

All the samples exhibit n‐type conduction as indicated by the negative Seebeck coefficient (*S*) over the full range of temperature (Figure 7b). Notably, this and our DFT results in Figure S4, Supporting Information, for the PbSe_0.8_Te_0.2_ sample clarify that its charge carriers mainly arise from the V_Te_ rather than V_Pb_ or V_Se_. Consequently, Te alloying promotes the formation of the V_Te_ in the PbSe_1−_ *_y_* Te*_y_* samples. In contrast, pristine PbSe without Te alloying,^[^ ^31^ ^]^ prepared by the same ball‐milling combined with SPS process as our samples, is reported to show the positive‐to‐negative sign crossover in the Seebeck coefficient at ≈650 K. This is possibly attributed to the easier formation of the V_Pb_ in Te‐free PbSe inducing p‐type conduction according to the previous defect formation energy calculation results.^[^ ^22a^ ^]^

Afterward, we theoretically calculated Pisarenko relation between |*S*| and *n*_H_ for pristine PbSe based on the assumption that acoustic phonon interaction is a principal scattering mechanism. The gray and red dashed lines are presented based on single parabolic band (SPB) model^[^ ^32^ ^]^ with density of states (DOS) effective mass *m*_0_ of 0.30 *m*_e_ at 300 K and *m*_0_ of 0.40 *m*_e_ at 723 K, respectively (Figure 7c). The experimental data for pristine PbSe^[^ ^17e^ ^]^ (black square), Pb_1−_ *_x_* Sb*_x_* Se^[^ ^17e^ ^]^ (navy circle), and PbSe_1−_ *_x_*_−_ *_y_* Te*_x_* Br*_y_* ^[^ ^25^ ^]^ (green diamond) from the previous reports are located near the theoretical Pisarenko lines for pristine PbSe both at 300 and 723 K. Remarkably, all the title Pb_1+_ *_x_* Se_0.8_Te_0.2_ (*x* = 0–0.125) samples in this work display much higher |*S*| at a given *n*_H_ at both 300 and 723 K. To understand the origin of the unusual increase in |*S*|, we prepared the control sample with the composition of Pb_1.075_Se_0.8_Te_0.2_ using traditional melt‐synthesis followed by SPS compaction (MS‐SPS). In striking contrast, its value (purple hexagon) falls closely on the gray dashed line along with pristine PbSe (blue square). Note that the main difference of the title samples is their much smaller grain size of ≈0.5 µm because of the ball‐milling process than ≈25 µm of the control sample. The increased |*S*| can be attributed to the change in charge scattering mechanism from acoustic phonon scattering at in‐grain regions for the control sample to ionized impurity scattering at grain boundaries for the title Pb_1+_ *_x_* Se_0.8_Te_0.2_ (*x* = 0–0.125) samples (Figure S6, Supporting Information). Similar effects have been published in various TE systems such as n‐ and p‐type PbTe^[^ ^33^ ^]^ and Bi_2_Te_3_.^[^ ^34^ ^]^ For example, the increased |*S*| is observed in I‐doped n‐type PbTe with the grain size decreasing less than ≈1.36 µm^[^ ^35^ ^]^ and InSb‐doped n‐type PbTe with the average grain size of ≈0.62 µm and multiscale in‐grain nanostructures.^[^ ^36^ ^]^

The highly unusual simultaneous increase in the |*S*|, *µ*_H_, and *n*_H_ uniquely decouple the core factors of thermoelectric figure of merit, ZT. First, |*S*| is unusually improved regardless of its intrinsic inverse proportionality to *σ*. Second, the simultaneous increase in the *n*_H_ and *µ*_H_ by excess Pb incorporation, contradicting to the general semiconductor physics, decouples the intrinsic inverse proportionality between *σ* and *S* in the specific range of *x*, giving the significantly increased *σ*. For example, the samples with *x* = 0.075 and 0.050 show the similar |*S*| of ≈130 µV K^−1^ even though the former exhibits ≈70% higher *σ* than the latter at 300 K.

These effects enable an extraordinary pathway to increasing power factor (PF). In fact, improving PF has been one of the most important tasks in thermoelectrics given no theoretical upper limit for PF. In contrast, thermal conductivity in many representative TE systems rapidly approaches a theoretical lower bound. Indeed, TE performance of p‐type PbSe has rapidly progressed mainly by virtue of availability in valence band convergence near the Fermi level, capable of increasing *S* without sacrificing *σ*.^[^ ^11^, ^17^, ^37^ ^]^ However, n‐type PbSe cannot benefit from similar strategies because of the single conduction band at the conduction band edge, lagging this technology behind its p‐type cousins. Very recently, PF of n‐type PbSe was improved by the introduction of new innovative strategies such as flattening conduction band edge^[^ ^12^, ^16^, ^19^, ^37^ ^]^ and increasing |*S*| by higher *m*_0_.^[^ ^13^, ^23^ ^]^ Our new strategies in this work demonstrate how to break intrinsic interrelation among the core factors comprising a TE figure merit and to improve overall performance by a chemical approach.

**Figure 8:**
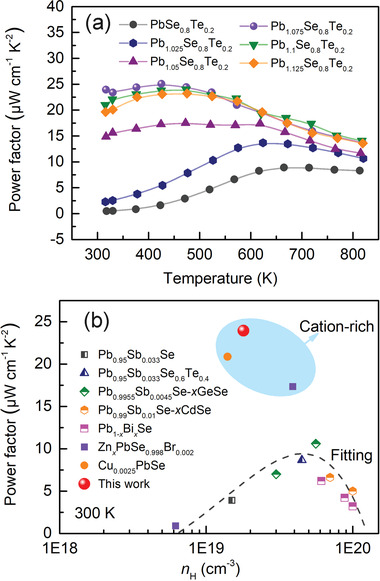
a) Temperature‐dependent power factor for the Pb_1+_ *_x_* Se_0.8_Te_0.2_ (*x* = 0–0.125) samples. b) The comparison of *n*_H_‐dependent power factor at 300 K of the Pb_1.075_Se_0.8_Te_0.2_ samples with those of the state‐of‐the‐art n‐type PbSe systems such as Pb_0.95_Sb_0.0033_Se_1−_ *_x_* Te*_x_*,^[^ ^10b^ ^]^ Pb_0.99_Sb_0.01_Se‐*x*CdSe,^[^ ^19^ ^]^ Pb_1−_ *_x_* Bi*_x_* Se,^[^ ^17e^ ^]^ Pb_0.9955_Sb_0.0045_Se‐*x*GeSe,^[^ ^17b^ ^]^ Zn_0.01_PbSe_0.998_Br_0.002_,^[^ ^22a^ ^]^ and Cu_0.0025_PbSe^[^ ^17d^ ^]^ from the previous reports.

The temperature‐dependent PF of the Pb_1+_ *_x_* Se_0.8_Te_0.2_ (*x* = 0–0.125) samples clearly shows the effect of excess Pb incorporation on the charge transport properties (**Figure** **8**a). It increases from 0.47 to 23.95 µW cm^−1^ K^−2^ at 300 K and from 8.26 to 13.71 µW cm^−1^ K^−2^ at 823 K as *x* increments from 0 to 0.075. Figure 8b compares the PF of the *x* = 0.075 sample with that of representative n‐type PbSe systems at a given *n*_H_ at 300 K, clearly differentiating the cation‐rich systems from the others. For example, Pb_0.95_Sb_0.0033_Se_1−_ *_x_* Te*_x_*,^[^ ^10b^ ^]^ Pb_0.99_Sb_0.01_Se‐*x*CdSe,^[^ ^19^ ^]^ Pb_1−_ *_x_* Bi*_x_* Se,^[^ ^17e^ ^]^ and Pb_0.9955_Sb_0.0045_Se‐*x*GeSe^[^ ^17b^ ^]^ show well‐fitted PF values to the classical model at 300 K. In contrast, the cation‐rich systems of Zn_0.01_PbSe_0.998_Br_0.002_,^[^ ^22a^ ^]^ Cu_0.0025_PbSe^[^ ^17d^ ^]^ and Pb_1.075_Se_0.8_Te_0.2_ give unusually high PF values of ≈21, 17 and 23.95 µW cm^−1^ K^−2^, respectively. The title system also shows remarkably high PF even from room to mid‐range temperatures, which is different from the common trend of PF for PbSe‐based materials. As a result, the *x* = 0.075 sample exhibits a significantly high average PF greater than 20 µW cm^−1^ K^−2^ from 300 to 823 K.

### 2.6 Thermal Transport Properties

**Figure 9:**
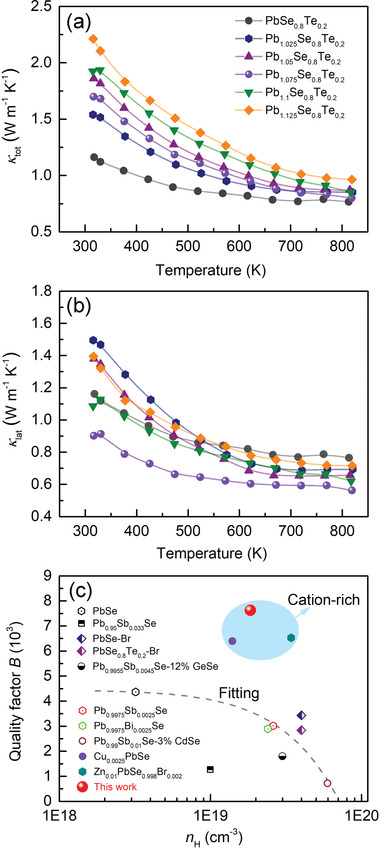
a) Total (*κ*_tot_) and b) lattice thermal conductivity (*κ*_lat_) of the Pb_1+_ *_x_* Se_0.8_Te_0.2_ samples (*x* = 0–0.125) with respect to temperature. c) The comparison of quality factor (*B*) of the title Pb_1.075_Se_0.8_Te_0.2_ sample with that of representative n‐type PbSe systems from the previous reports such as PbSe,^[^ ^17e^ ^]^ Pb_0.9975_Sb_0.0025_Se,^[^ ^17e^ ^]^ Pb_0.9975_Bi_0.0025_Se,^[^ ^17e^ ^]^ Pb_0.9955_Sb_0.0045_Se‐12%GeSe,^[^ ^17b^ ^]^ Pb_0.99_Sb_0.01_Se‐3%CdSe,^[^ ^19^ ^]^ PbSe_1−_ *_x_* Te*_x_*‐Br (*x* = 0 and 0.2),^[^ ^25^ ^]^ Zn_0.01_PbSe_0.998_Br_0.002_,^[^ ^22a^ ^]^ Cu_0.0025_PbSe,^[^ ^17d^ ^]^ and Pb_0.95_Sb_0.033_Se^[^ ^30^ ^]^ at 300 K. The gray dashed line is fitted based on the data from the previous works for PbSe,^[^ ^17e^ ^]^ Pb_0.9975_Sb_0.0025_Se,^[^ ^17e^ ^]^ Pb_0.9975_Bi_0.0025_Se,^[^ ^17e^ ^]^ Pb_0.9955_Sb_0.0045_Se‐12%GeSe,^[^ ^17b^ ^]^ Pb_0.99_Sb_0.01_Se‐3%CdSe^[^ ^19^ ^]^ and PbSe_1−_ *_x_* Te*_x_*‐Br (*x* = 0 and 0.2),^[^ ^25^ ^]^ clearly showing the highest decoupling for *µ*_H_ and *κ*_lat_ in the Pb_1.075_Se_0.8_Te_0.2_ sample.

The temperature‐dependent total thermal conductivity (*κ*_tot_) for the Pb_1+_ *_x_* Se_0.8_Te_0.2_ samples (*x* = 0–0.125) increases with greater *x* (**Figure** **9**a) due to the increased electrical contribution to it. However, the gap between *κ*_tot_ values with respect to *x* rapidly diminishes with increasing temperature. For example, the *κ*_tot_ for the *x* = 0 and 0.075 samples are 1.21 and 1.69 W m^−1^ K^−1^ at 300 K and 0.77 and 0.80 W m^−1^ K^−1^ at 823 K.

The lattice thermal conductivity (*κ*_lat_) is obtained by subtracting electrical thermal conductivity (*κ*_ele_) from *κ*_tot_ using Wiedemann–Franz relationship^[^ ^32a^ ^]^ (see Figure S7, Supporting Information, for *κ*_ele_). The *κ*_lat_ decreases with increasing temperature because of Umklapp scattering^[^ ^38^ ^]^ (Figure 9b). It is depressed significantly by phonon scattering due to the presence of Pb_Te_ antisite defects and elastic distortions of the crystal lattice induced by excess Pb atoms as observed in our STEM results. The *x* = 0.075 sample shows the lowest *κ*_lat_ among the series over the entire temperature range: ≈0.90 and 0.60 W m^−1^ K^−1^ at 300 and 823 K, respectively. This trend possibly arises from the combined effect of the following contrasting attributes in the Pb_1+_ *_x_* Se_0.8_Te_0.2_ samples. Pb‐rich nanostructures can reduce *κ*_lat_ by scattering phonon.^[^ ^10a^ ^]^ On the other hand, the increased content of Pb precipitates can also raise *κ*_lat_ because of a much higher *κ*_tot_ of Pb metal (34 W m^−1^ K^−1^ at 300 K^[^ ^39^ ^]^) than the matrix phase.

### 2.7 Decoupling Charge and Thermal Transport Properties

Defects in solids affect charge and thermal transport simultaneously. To achieve maximal TE performance of materials, a prime interest in thermoelectrics has been developing effective strategies to decouple close interrelationship between carrier mobility (*µ*_H_) and *κ*_lat_. Accordingly, a greater ratio of *µ*_H_ to *κ*_lat_ is a key parameter to optimize TE materials, which is typically expressed by quality factor *B*.^[^ ^5^, ^40^ ^]^ The *x* = 0.075 sample shows record‐high *B* value of ≈7.6 × 10^3^ at 300 K, far greater than that of the reported state‐of‐the‐art n‐type PbSe systems (Figure 9c).^[^ ^17^, ^25^, ^30^ ^]^ The dashed line is given for easy comparison for *B* values, which is fitted based on experimental data of pristine PbSe,^[^ ^17e^ ^]^ Pb_0.9975_Sb_0.0025_Se,^[^ ^17e^ ^]^ Pb_0.9975_Bi_0.0025_Se,^[^ ^17e^ ^]^ Pb_0.9955_Sb_0.0045_Se‐12%GeSe,^[^ ^17b^ ^]^ Pb_0.99_Sb_0.01_Se‐3%CdSe and PbSe_1−_ *_x_* Te*_x_*‐Br (*x* = 0 and 0.2).^[^ ^25^ ^]^ The cation‐rich PbSe systems such as Zn_0.01_PbSe_0.998_Br_0.002_,^[^ ^22a^ ^]^ Cu_0.0025_PbSe,^[^ ^17d^ ^]^ and the title material Pb_1.075_Se_0.8_Te_0.2_ give unusually boosted *B* values. In contrast, Pb_0.95_Sb_0.033_Se,^[^ ^30^ ^]^ with a high concentration vacancy, shows the much lower *B* value.

### 2.8 Thermoelectric Performance

**Figure 10:**
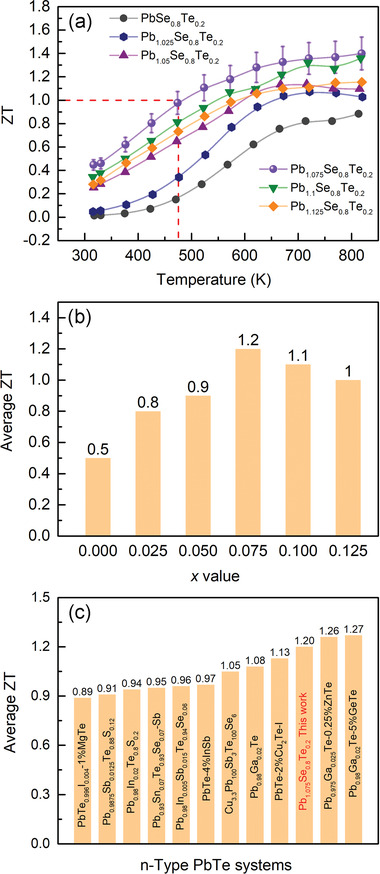
a) Thermoelectric figure of merit, ZT, as a function of temperature and b) average ZT (ZT_ave_) from 400 to 823 K for the Pb_1+_ *_x_* Se_0.8_Te_0.2_ (*x* = 0–0.125) samples. c) The comparison of the ZT_ave_ of the Pb_1.075_Se_0.8_Te_0.2_ sample with the top‐performing n‐type PbTe thermoelectric systems such as PbTe_0.996_I_0.004_‐1%MgTe,^[^ ^33d^ ^]^ Pb_0.9875_Sb_0.0125_Te_0.88_S_0.12_,^[^ ^45^ ^]^ Pb_0.98_In_0.02_Te_0.8_S_0.2_,^[^ ^46^ ^]^ Pb_0.93_Sn_0.07_Te_0.93_Se_0.07_‐Sb,^[^ ^47^ ^]^ Pb_0.98_In_0.005_Sb_0.015_Te_0.94_Se_0.06_,^[^ ^48^ ^]^ PbTe‐4%InSb,^[^ ^36^ ^]^ Cu_3.3_Pb_100_Sb_3_Te_100_Se_6_,^[^ ^20^ ^]^ Pb_0.98_Ga_0.02_Te,^[^ ^49^ ^]^ PbTe‐2%Cu_2_Te‐I,^[^ ^22b^ ^]^ Pb_0.975_Ga_0.025_Te‐0.25%ZnTe,^[^ ^21^ ^]^ and Pb_0.98_Ga_0.02_Te‐5%GeTe.^[^ ^50^ ^]^

**Figure** **10**a shows temperature‐dependent TE figures of merit, ZT, for the Pb_1+_ *_x_* Se_0.8_Te_0.2_ system (*x* = 0–0.125). The *x* = 0.075 sample shows a highest peak ZT (ZT_max_) of 1.40 at 823 K among the series. Remarkably, it exhibits a high ZT of ≈0.45 even at 300 K and surpasses unity above ≈473 K as indicated by the red broken line in Figure 10b, which is rarely achieved by other PbSe‐based materials. This observation demonstrates that TE performance at low and intermediate temperature ranges can also be improved considerably by defect engineering.

For practical applications of TE technology, average ZT (ZT_ave_) is more important than ZT_max_ for stable TE power generation over a wide range of temperature.^[^ ^41^ ^]^ However, most TE materials give a high ZT in a very narrow range of temperature. The ZT_ave_ can be typically expressed by Equation (5):^[^ ^42^ ^]^

(5) $${\mathrm{ZT}}_{\mathrm{ave}}=\int_{T_{C}}^{T_{H}}\frac{\mathrm{ZT}}{T_{H}-T_{C}}dT$$

The *x* = 0.075 sample exhibits a highest ZT_ave_ of ≈1.20 from 400 to 823 K among the series (Figure 10b). In comparison, the ZT_ave_ for the state‐of‐the‐art n‐type PbSe‐based thermoelectric systems in a similar temperature range is ≈0.87 for Pb_0.95_Sb_0.033_Se,^[^ ^30^ ^]^ ≈1.16 for Cu_0.00375_PbSe,^[^ ^17d^ ^]^ ≈1.1 for PbSe_0.998_Br_0.002_‐2%Cu_2_Se,^[^ ^12a^ ^]^ ≈1.04 for Pb_0.94_In_0.02_Se,^[^ ^43^ ^]^ ≈0.94 for Zn_0.01_PbSe_0.998_Br_0.002_,^[^ ^22a^ ^]^ ≈0.77 for Pb_0.99_Sb_0.01_Se‐3%CdSe,^[^ ^19^ ^]^ ≈1.3 for Cu_0.005_PbSe_0.99_Te_0.01_,^[^ ^13b^ ^]^ and ≈1.18 for Pb_0.89_Sb_0.012_Sn_0.1_Se_0.5_Te_0.25_S_0.25_.^[^ ^11b^ ^]^ The obtained value is exceedingly higher than the highest ZT_ave_ reported for p‐type PbSe materials, for example, ≈0.62 for Pb_0.92_Sr_0.08_Se,^[^ ^44^ ^]^ ≈0.41 for Pb_0.98_Na_0.02_Se‐2%HgSe,^[^ ^17a^ ^]^ ≈0.83 for Pb_0.98_K_0.02_Se‐6%CdSe^[^ ^17c^ ^]^ and ≈1 for Pb_0.95_Na_0.02_Cd_0.03_Se_0.85_Te_0.15_.^[^ ^11a^ ^]^ Importantly, the achieved ZT_ave_ is even higher than many state‐of‐the‐art n‐type PbTe‐based thermoelectric materials in a similar temperature range (Figure 10c), for instance, ≈0.89 for PbTe_0.996_I_0.004_‐1%MgTe,^[^ ^33d^ ^]^ ≈0.91 for Pb_0.9875_Sb_0.0125_Te_0.88_S_0.12_,^[^ ^45^ ^]^ ≈0.94 for Pb_0.98_In_0.02_Te_0.8_S_0.2_,^[^ ^46^ ^]^ ≈0.95 for P_b0.93_Sn_0.07_Te_0.93_Se_0.07_‐Sb,^[^ ^47^ ^]^ ≈0.96 for Pb_0.98_In_0.005_Sb_0.015_Te_0.94_Se_0.06_,^[^ ^48^ ^]^ ≈0.97 for PbTe‐4%InSb,^[^ ^36^ ^]^ ≈1.05 for Cu_3.3_Pb_100_Sb_3_Te_100_Se_6_,^[^ ^20^ ^]^ ≈1.08 for Pb_0.98_Ga_0.02_Te^[^ ^49^ ^]^ and ≈1.13 for PbTe‐2%Cu_2_Te‐I.^[^ ^22b^ ^]^ It is even comparable to the record‐high ZT_ave_ reported for the n‐type PbTe system: ≈1.26 and ≈1.27 for Pb_0.975_Ga_0.025_Te‐0.25%ZnTe^[^ ^21^ ^]^ and Pb_0.98_Ga_0.02_Te‐5%GeTe,^[^ ^50^ ^]^ respectively.

The nonstoichiometric, Pb excess composition of the Pb_1.075_Se_0.8_Te_0.2_ sample does not deteriorate the TE performance on the continuous heating and cooling cycles. Its *σ*, *S*, *κ*_tot_, *κ*_lat_ and PF during a round‐trip cycle show a negligible hysteresis within the instrumental errors, verifying the reversibility of its performance on the repeated heating cycles (Figure S8, Supporting Information). The reproducibility and reliability of its TE properties were cross‐checked in two different institutions of Seoul National University and Tsinghua University using the independent samples.

We presented here that hardly unavoidable physical restrictions in a TE figure of merit, ZT, can be broken by chemical alloying and excessive doping, coupled with a mechanical treatment of reducing crystallite size. They simultaneously improve the mobility and concentration of charge carrier; and electrical conductivity and Seebeck coefficient at the specific concentration range unconventionally. They consequently circumvent the intrinsic disadvantage of n‐type PbSe‐based materials, namely, single conduction band inhibiting the convergence of two neighboring bands near Fermi level. Notably, the observed TE properties with respect to temperature trend very differently from typical PbSe materials, as dramatically observed in the exceptionally high ZT of ≈0.45 at 300 K for TE systems operating in the intermediate temperature range. Eventually, much cheaper n‐type PbSe rivals its telluride analogue in average ZT, greatly escalating the prospect of practical applications of TE power generation.

Very importantly, our defect architecture, ranging from atomic to nanoscale to submicron scale, is naturally created thermodynamically driven by the Te alloying and excess Pb incorporation, thereby mass‐producible, thermally stable, and highly reproducible as experimentally confirmed. Our design principle, defect formation mechanism, and direct observation of atomic resolution defect structures help to understand how defects form in the crystal matrix at the atomic level and to build the foundation for more predictable, tailor‐made design for functional materials. The achievement in this work highlights the importance of exploring available chemical compositions and their ranges in the representative TE systems.

## 3 Experimental Section

### Synthesis

An appropriate molar ratio of Pb powder (99.95%, Aladdin), Se powder (99.999%, Aladdin), and Te powder (99.999%, Aladdin) was loaded in an evacuated steel jar and milled with steel balls for 2 h using a 3D vibration HEBM machine. The resulting powder was loaded in an alloy mold and sintered by a SPS instrument (Ed‐PASIII, Elenix Ltd.) in a vacuum under a pressure of 50 MPa at 823 K for 5 min. The obtained SPS‐processed samples showed the typical theoretical density greater than 95% (Table S1, Supporting Information) and dimension of Φ10 mm × 15 mm.

### Physical Characterization

PXRD patterns were recorded on a SmartLab Rigaku powder X‐ray diffractometer using Cu K*α* (*λ* = 1.5418 Å) graphite‐monochromatized radiation at 40 kV and 30 mA. They were analyzed using a Jade 6.5 software tool. The microstructure, elemental distribution, and chemical compositions were examined by a scanning electron microscope (SEM, Zeiss Gemini SEM 500) equipped with an energy‐dispersive spectroscopy (EDS). The orientation and size of crystallites were observed by electron backscatter diffraction (EBSD) from a SEM (Zeiss Gemini SEM SU3500). In situ temperature‐dependent PXRD patterns were collected on an X'Pert Pro MPD from 300 to 698 K. The TGA and DSC curves were obtained by a thermal gravimetric analyzer (TG 209, Netzsch) and DSC (DSC 214, Netzsch) under an Ar flow at a heating rate of 10 K min^−1^.

### Atom Probe Tomography (APT)

The APT specimens were prepared by the site‐specific “lift‐out” method^[^ ^51^ ^]^ employing a focused ion beam (FIB) (Helio NanoLab 650, FEI) with a dual‐beam SEM. The specimens were examined in a local electrode atom probe (LEAP 4000 X Si, Cameca) with 10 ps and 30 pJ ultraviolet (*λ* = 355 nm) laser pulses at a pulse repetition rate of ≈200 kHz.

### Transmission Electron Microscopy (TEM)

For cross‐sectional STEM observation, SPS‐processed samples were thinned down to cross‐sectional wedges by gallium ion milling from a focused ion beam (FIB) equipped with a dual beam microscope (Helios G4, Thermo Scientific). The local structures and compositions were investigated by a spherical aberration‐corrected JEM ARM‐200F microscope (Cold FEG Type, JEOL). To record high‐angle annular dark‐field (HAADF)‐STEM images, the resolution was ≈80 pm after the spherical aberration‐correction and the angular range of the annular detector was from 68 to 280 mrad. All STEM images were taken by a charge coupled device (CCD) detector with a high‐resolution 2k × 2k pixel device in the GIF‐QuantumER imaging filter (GATAN). For the STEM‐EDS data, the elements maps were scanned with a probe size of 0.13 nm and a probe current of 40 pA by a silicon drift detector (SDD) type EDS detector (Solid Angle 0.9‐sr, X‐MaxN 100TLE, OXFORD) at 200 kV. All TEM works were conducted at the National Center for Inter‐University Research Facilities (NCIRF) in Seoul National University.

### Charge Carrier Transport

SPS‐processed samples were cut and polished into appropriate shapes and dimensions to measure charge and thermal transport properties. The bar‐shaped samples with the dimension of ≈10 × 2 × 3 mm^3^ were used to measure Seebeck coefficient (*S*) and electrical conductivity (*σ*) concurrently by an ULVAC‐RIKO ZEM‐3 instrument under a low‐pressure He atmosphere from room temperature to 823 K. Hall effect measurements as a function of temperature were conducted on a Lakeshore 8407 system from 300 to 823 K under an Ar flow atmosphere with a reversible 1.5 T magnetic field and 5 mA excitation current.

### Thermal Conductivity

Temperature‐variant thermal diffusivity was measured for disks with a diameter of ≈6 mm and a thickness of ≈1.5 mm using the laser flash diffusivity method on a Netzsch LFA 457 instrument under an Ar flow. The surface of specimens was protected by a graphite coating. The thermal conductivity (*κ*_tot_) was calculated according to the relation *κ*_tot_ = *D C*_p_ *ρ*, where *D* is the measured thermal diffusivity, *C*_p_ is the specific heat capacity, and *ρ* is the density. *C*_p_ was calculated from the relation *C*_p_/*k*_B_ per atom = 3.07 + [4.7 × 10^−4^ × (*T* − 300)]^[^ ^11a^ ^]^ and *ρ* was obtained from the geometrical dimensions and masses of the samples (Table S1, Supporting Information). The lattice thermal conductivity (*κ*_lat_) was obtained by the relation *κ*_lat_ = *κ*_tot_ − *κ*_ele_ (*κ*_e_ = *L σ T*), where the *L* is Lorenz number calculated with *S* and *σ* by single parabolic band (SPB) model^[^ ^32^ ^]^ as described in the Supporting Information.

### Density Functional Theory (DFT) Calculations

Theoretical calculations at the DFT level were calculated using a Cambridge Sequential Total Energy Package (CASTEP) and the generalized gradient approximation (GGA) within the Perdew–Burke–Ernzerhof (PBE) formulation.^[^ ^18^ ^]^ A plane wave cutoff energy of 700 eV was used in all DFT calculations. The *k*‐point of the crystal structure was set as 4 × 4 × 4. The self‐consistent field (SCF) tolerance was set at 2.0 × 10^−6^ eV per atom. A preliminary 2 × 2 × 2 PbSe supercell containing 64 atoms was used to simulate the authors’ systems. Isovalent Group 16 congener Te was allocated to the Se site. All the atoms in the supercell were optimized until the geometric structure reached the forces on every atom to be less than 0.05 eV Å^−1^, their total energy difference less than 2 × 10^−5^ eV, the maximum ionic placement less than 0.002 Å, and the maximum stress less than 0.1 GPa.

## Conflict of Interest

The authors declare no conflict of interest.

## Supporting information

Supporting Information

## Data Availability

The data that support the findings of this study are available in the supplementary material of this article.
