# Supplementary material for: Atomic Level Defect Structure Engineering for Unusually High Average Thermoelectric Figure of Merit in n‐Type PbSe Rivalling PbTe
Source: Adv Sci (Weinh). 2022 Oct 26;9(35):2203782. doi: 10.1002/advs.202203782 (PMC9762289; doi:10.1002/advs.202203782)
Supplement: Supplementary file 1 — Supporting Information [file ADVS-9-2203782-s001.pdf]

ADVANCED SCIENCE
Open Access

Supporting Information

for *Adv. Sci.*, DOI 10.1002/advs.202203782

Atomic Level Defect Structure Engineering for Unusually High Average Thermoelectric Figure of Merit in n-Type PbSe Rivalling PbTe

Bangzhi Ge, Hyungseok Lee, Lulu Huang, Chongjian Zhou, Zhilei Wei, Bowen Cai, Sung-Pyo Cho, Jing-Feng Li, Guanjun Qiao, Xiaoying Qin, Zhongqi Shi and In Chung**

Supporting information:

Atomic Level Defect Structure Engineering for Unusually High Average Thermoelectric Figure of Merit in n-Type PbSe Rivalling PbTe

Bangzhi Ge, Hyungseok Lee, Lulu Huang, Chongjian Zhou, Zhilei Wei, Bowen Cai, Sung-Pyo Cho, Jing-Feng Li, Guanjun Qiao, Xiaoying Qin, Zhongqi Shi,* and In Chung*

Mr. B. Ge, Mr. Z. Wei, Prof. G. Qiao, and Prof. Z. Shi

State Key Laboratory for Mechanical Behavior of Materials, Xi'an Jiaotong University, Xi'an 710049, China

Email: zhongqishi@mail.xjtu.edu.cn

Mr. B. Ge, Mr. H. Lee, Dr. L. Huang, Dr. C. Zhou, and Prof. I. Chung

School of Chemical and Biological Engineering and Institute of Chemical Processes, Seoul National University, Seoul 08826, Republic of Korea

Email: inchung@snu.ac.kr

Prof. S. -P. Cho

National Center for Inter-University Research Facilities, Seoul National University, Seoul 08826, Republic of Korea

Mr. H. Lee and Prof. I. Chung

Center for Correlated Electron Systems, Institute for Basic Science (IBS), Seoul 08826, Republic of Korea

Dr. L. Huang and Prof. X. Qin

Key Lab of Photovoltaic and Energy Conservation Materials, Institute of Solid State Physics, HFIPS, Chinese Academy of Sciences, Hefei 230031, China

Dr. B. Cai and Prof. J. -F Li

State Key Laboratory of New Ceramics and Fine Processing, School of Materials Science and Engineering, Tsinghua University, Beijing 100190, China

Prof. G. Qiao

School of Materials Science and Engineering, Jiangsu University, Zhenjiang 212013, China

*To whom correspondence should be addressed: inchung@snu.ac.kr, zhongqishi@mail.xjtu.edu.cn

Content

1. Details in the Theoretical Calculations

2. Supporting Tables

Table S1. Density of the $Pb_{1+x}Se_{0.8}Te_{0.2}$ samples after spark plasma sintering (SPS) process (x = 0 – 0.075)

Table S2. Quantitative elemental analysis by STEM-EDS taken at the matrix and nanostructure embedded in the $Pb_{1.075}Se_{0.8}Te_{0.2}$ sample. A relative molar ratio for Pb, Se, and Te are given. Although STEM-EDS does not provide the exact value, it is enough to reveal Pb-rich regions.

3. Supporting Figures

Figure S1. The backscattered electron image of the SPS processed $Pb_{1.075}Se_{0.8}Te_{0.2}$ sample. The white Pb precipitates are embedded within the matrix as indicated by red arrows.

Figure S2. The electron backscatter diffraction image for the control $Pb_{1.075}Se_{0.8}Te_{0.2}$ sample, which was prepared by traditional melt synthesis followed by SPS process.

Figure S3. (a) Medium-magnification ABF-STEM image of the $Pb_{1.075}Se_{0.8}Te_{0.2}$ sample in Figure 5a. (b) fast Fourier transform image (FFT) taken at the surrounding matrix and (c) lattice distortion area. The both display a single set of the patterns corresponding to the rock-salt structure down to the <110> zone axis. .

Figure S4. Defect formation energy calculated as a function of Fermi energy for the $Pb_{32}Se_{24}Te_8$ supercell under both the (a) Pb-rich and (b) Pb-poor conditions.

Figure S5. Thermal behavior of the $Pb_{1.075}Se_{0.8}Te_{0.2}$ sample. (a) The in-situ temperature-dependent PXRD patterns. The characteristic Bragg peak of elemental Pb around 31° is magnified in the right side of the panel. (b) The refined lattice parameters with respect to temperature. (c) Thermogravimetric analysis under an Ar flow showing its thermal stability. (d) Differential scanning calorimetry curves for the $Pb_{1.075}Se_{0.8}Te_{0.2}$ sample and pure Pb reference upon the consecutive heating and cooling cycles.

Figure S6. Theoretical Pisarenko relation between the magnitude of S ($|S|$) and n_H calculated based on single parabolic band (SPB) model at 300 K. The theoretical Pisarenko line with the acoustic phonon scattering (r = –1/2) mechanism and density of states effective mass (m_0) of 0.30 m_e is presented as the gray line. The $|S|$ of pristine PbSe[1] and the control sample $Pb_{1.075}Se_{0.8}Te_{0.2}$ prepared by traditional melt-synthesis followed by SPS process (purple circle) are closely located at this line. In contrast, the experimental $|S|$ values of the title $Pb_{1+x}Se_{0.8}Te_{0.2}$ (x = 0 − 0.125) samples lie far above this line and move towards red line (r = 3/2) representing ionized impurity scattering model.

Figure S7. The electronic thermal conductivity (κ_{ele}) of the $Pb_{1+x}Se_{0.8}Te_{0.2}$ samples (x = 0 – 0.125) with respect to temperature.

Figure S8. The (a) electrical conductivity (σ), (b) Seebeck coefficient (S), (c) thermal conductivity (κ), (d) power factor (PF), (e) lattice thermal conductivity (κ_{lat}), and (e) thermoelectric figure of merit (ZT) of the $Pb_{1.075}Se_{0.8}Te_{0.2}$ sample. The sample 1 was measured for the consecutive heating and cooling cycle in Seoul National University of Republic of Korea, demonstrating cyclability and thermal stability of the material for thermoelectric power generation in a wide range of temperature. The independently synthesized sample 2 was characterized in Tsinghua University, China, confirming the reproducibility and reliability of thermoelectric performance of the $Pb_{1.075}Se_{0.8}Te_{0.2}$ sample.

4. References

1. Details in the Theoretical Calculations

Single parabolic band (SPB) model[2]. Because PbQ (Q=Se and Te) shows single band nature at the conduction band, SPB model was used to calculate the effective mass of electron (m_0) and Lorenz number (L).[1] Assuming SPB model, the Seebeck coefficient (S), the nth order Fermi integral ($F_n(\eta)$), and the m_0 were computed using the equations S1-S4:

$$S = \pm\frac{k_B}{e}\left(\frac{(5/2+r)F_{3/2+r}(\eta))}{(3/2+r)F_{1/2+r}(\eta))} - \eta\right) \tag{R1}$$

$$F_n(\eta) = \int_0^\infty \frac{\chi^n}{1+e^{\chi-\eta}}d\chi \tag{R2}$$

$$r_H = \frac{3}{2}\frac{(3/2+2r)F_{1/2}(\eta)F_{2r+1/2}(\eta)}{(3/2+r)^2F_{r+1/2}^2(\eta)} \tag{R3}$$

$$m_0 = \frac{h^2}{2k_BT}\left[\frac{n\cdot r_H}{4\pi F_{1/2}(\eta)}\right]^{2/3} \tag{R4}$$

where η is the reduced Fermi energy, e is the charge of an electron, r_H is the Hall factor, h is the Planck constant, k_B is the Boltzmann constant, T is the absolute temperature, and r is the scattering parament. The r is set at –1/2, 1/2, 3/2, and 0 when dominant charge scattering mechanism is acoustic phonon, optical phonon, ionized impurity, and neutral impurity scattering, respectively.

The results of temperature-dependent Hall carrier mobility (μ_H) with respect to temperature in Figure 6a in the main text indicate that the samples with x = 0 – 0.025 show the vacancy scattering mechanism and those with x = 0.05 – 0.125 follow lattice scattering mechanism. In SPB model, the former and latter correspond to the scattering mechanism dominated by acoustic phonon (r = –1/2) and ionized impurity (r = 3/2), respectively. We calculated Pisarenko relation assuming the aforementioned scattering mechanisms in Figure S6. The gray and red lines are given based on acoustic phonon and ionized impurity scattering mechanisms, respectively, with m_0 of

$0.30m_e$ at 300 K. The experimental S values at the given n_H of the $Pb_{1+x}Se_{0.8}Te_{0.2}$ (x = 0 − 0.125) samples significantly deviate from the ionized impurity scattering mechanism model and moves toward acoustic phonon scattering mechanism model with the introduction of excess Pb (green arrow in Figure S6), which agrees with the findings in Figure 6a.

According to the previous report,[3] charge is scattered at in-grain regions and grain boundaries according to acoustic phonon and ionized impurity scattering mechanisms, respectively. The experimental Seebeck coefficients of pristine PbSe (black square)[1] and our control sample $Pb_{1.075}Se_{0.8}Te_{0.2}$ prepared by traditional melt-synthesis followed by SPS process (purple circle) fall well on the gray line. In sharp contrast, the title ball milled $Pb_{1.075}Se_{0.8}Te_{0.2}$ sample shows their Seebeck coefficients lying far above the gray line and rather moving toward the red line (purple arrow in Figure S6). Namely, grain boundary affects charge transport and thus enhances the Seebeck coefficient due to ionized impurity scattering.

Because thermal conductivity (κ) is contributed by electronic κ_e and lattice κ_{lat} thermal conductivity, the subtraction of κ_{ele} from κ is calculated by Wiedeman-Franz relation: $\kappa_{lat} = \kappa - \kappa_{ele}$ ($\kappa_{ele} = L\,\sigma\,T$),[4] where σ and L is electrical conductivity and Lorenz number, respectively. L is calculated using the equation[5] (S5), combined with equations (S1) and (S3).

$$L = \left(\frac{k_B}{e}\right)^2 \left[\frac{(r+\frac{7}{2})F_{r+5/2}(\eta)}{(r+\frac{3}{2})F_{r+1/2}(\eta)} - \frac{(r+\frac{5}{2})F_{r+3/2}(\eta)}{(r+\frac{3}{2})F_{r+1/2}(\eta)}\right] \quad \text{(S5)}$$

where r is the scattering factor (r = 1/2).

Density Functional Theory (DFT) calculations. Theoretical calculations at the DFT

level were conducted using a Cambridge Sequential Total Energy Package (CASTEP) and the generalized gradient approximation (GGA) within the Perdew-Burke-Ernzerhof (PBE) formulation.[6] A plane wave cutoff energy of 700 eV was used in all DFT calculations. The k-point of the crystal structure was set at 4 × 4 × 4. The self-consistent field (SCF) tolerance was used at 2.0 × 10^{-6} eV per atom. A preliminary 2 × 2 × 2 PbSe supercell containing 64 atoms was used to simulate our system. The isovalent Group 16 congener Te was allocated to the crystallographic Se site. All the atoms in the supercell were optimized until the geometric structure reached the forces on every atom less than 0.05 eV $Å^{-1}$, their total energy difference less than 2 × 10^{-5} eV, the maximum ionic placement less than 0.002 Å, and the maximum stress less than 0.1 GPa.

The expression for the formation energy ($\Delta H_{d,q}$) of defect (d) in the charge state (q) is defined by the equation (S6):[7]

$$\Delta H_{d,q}(E_{F},\mu) = E_{d,q} - E_{p} - \Sigma n_{\alpha}\mu_{\alpha} + q(E_{F} + E_{V} + \varepsilon) \quad \text{(S6)}$$

where $E_{d,q}$ and E_{p} are the total energies of the supercell with the defects obtain from CASTEP in the q and a perfect host supercell, respectively. n_{α} is the number of exchanged atoms (α) in defect supercell system, and μ_{α} is the corresponding chemical potential of α. E_{F} is the Fermi level, and E_{V} corresponds to the valence band maximum, which was corrected by ε. The formation energy of the defects is a function of the E_{F} and μ_{α} of reactants.

In order to give the relationship between $\Delta H_{d,q}(E_{F},\mu)$ and E_{F}, the boundary conditions of μ_{α} need to be given. Based on the thermodynamic limits on the

achievable values of the chemical potentials,[7b] the μ_{α} can be obtained in Pb-rich and Pb-poor conditions by the method described in previous work.[7b] In Pb-rich condition, the μ_{Pb} = 0 eV, μ_{Se} = ΔE_{PbSe} = –1.08 eV and μ_{Te} =ΔE_{PbTe}= –0.88 eV. In Pb-poor condition, μ_{Pb} = E_{PbSe} = –1.08 eV, μ_{Se} = 0 eV and μ_{Te} = 0 eV. Based on the above results, we can obtain the relationship between the $\Delta H_{d,q}(E_{F},_{\mu})$ and the E_{F} in Pb-rich and Pb-poor conditions.

The formation energies of PbQ (ΔE_{PbQ}, Q = Se, Te) were calculated by the following relation:

$$\Delta E_{PbQ} = E_{PbQ} - n_{Pb}E_{Pb} - n_{Q}E_{Q} \quad \text{(S7)}$$

where E_{PbQ}, E_{Pb} and E_{Q} are the total energies of the PbQ supercell, Pb atom and Q atom obtain from CASTEP, respectively. n_{Pb} and n_{Q} are the number of Pb and Q in the PbQ supercell, respectively.

2. Supporting Table

Table S1. Density of the $Pb_{1+x}Se_{0.8}Te_{0.2}$ samples after spark plasma sintering (SPS) process (x = 0 – 0.075).

Samples	Density (g cm^{-3})	Relative density (%)
$PbSe_{0.8}Te_{0.2}$	7.85	95.73
$Pb_{1.025}Se_{0.8}Te_{0.2}$	7.88	96.10
$Pb_{1.05}Se_{0.8}Te_{0.2}$	7.87	96.00
$Pb_{1.075}Se_{0.8}Te_{0.2}$	7.90	96.34
$Pb_{1.1}Se_{0.8}Te_{0.2}$	7.92	96.59
$Pb_{1.125}Se_{0.8}Te_{0.2}$	7.92	96.59

Table S2. Quantitative elemental analysis by STEM-EDS taken at the matrix and nanostructure embedded in the $Pb_{1.075}Se_{0.8}Te_{0.2}$ sample. A relative molar ratio for Pb, Se, and Te are given. Although STEM-EDS does not provide the exact value, it is enough to reveal Pb-rich regions.

Region	Pb at.%	Se at.%	Te at.%
Matrix 1	49.22	39.56	11.22
Matrix 2	49.59	39.34	11.07
Nanostructure 1	51.06	40.05	8.89
Nanostructure 2	51.25	39.67	9.08
Nanostructure 3	51.16	39.71	9.13

3. Supporting Figures

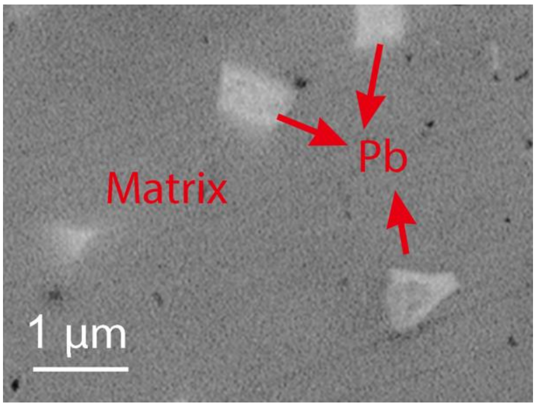

Figure S1. The backscattered electron image of the SPS processed $Pb_{1.075}Se_{0.8}Te_{0.2}$ sample. The white Pb precipitates are embedded within the matrix as indicated by red arrows.

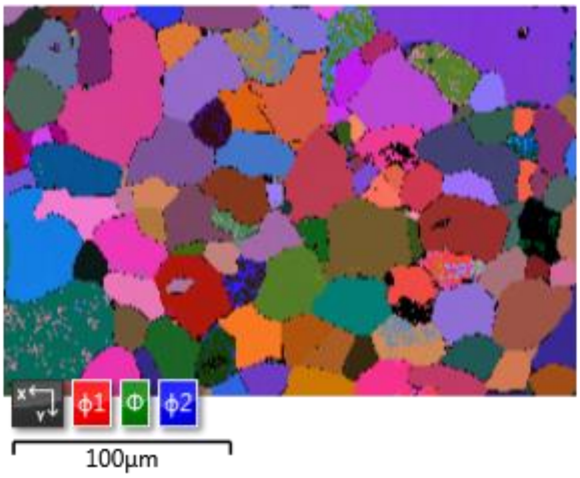

Figure S2. The electron backscatter diffraction image for the control $Pb_{1.075}Se_{0.8}Te_{0.2}$ sample, which was prepared by traditional melt synthesis followed by SPS process.

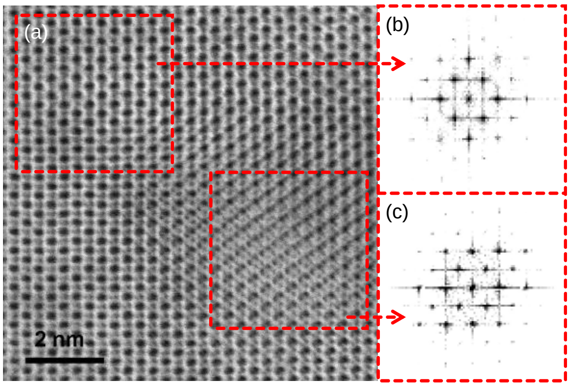

Figure S3. (a) Medium-magnification ABF-STEM image of the $Pb_{1.075}Se_{0.8}Te_{0.2}$ sample in Figure 5a. (b) fast Fourier transform image (FFT) taken at the surrounding matrix and (c) lattice distortion area. The both display a single set of the patterns corresponding to the rock-salt structure down to the <110> zone axis.

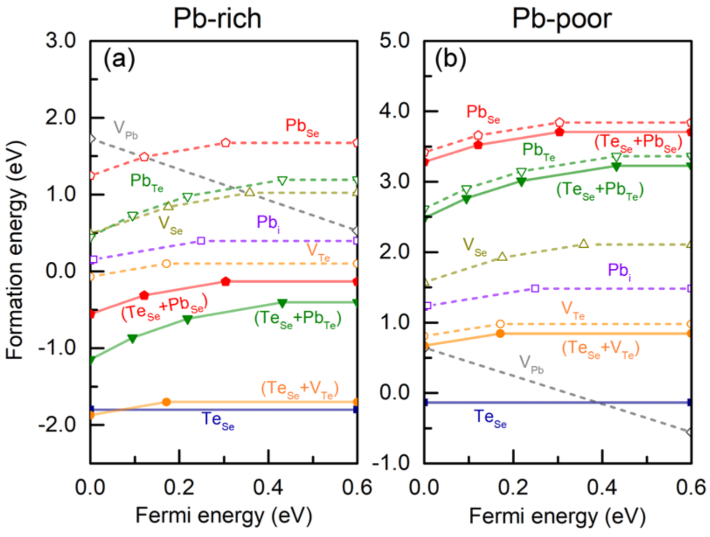

Figure S4. Defect formation energy calculated as a function of Fermi energy for the $Pb_{32}Se_{24}Te_8$ supercell under both the (a) Pb-rich and (b) Pb-poor conditions.

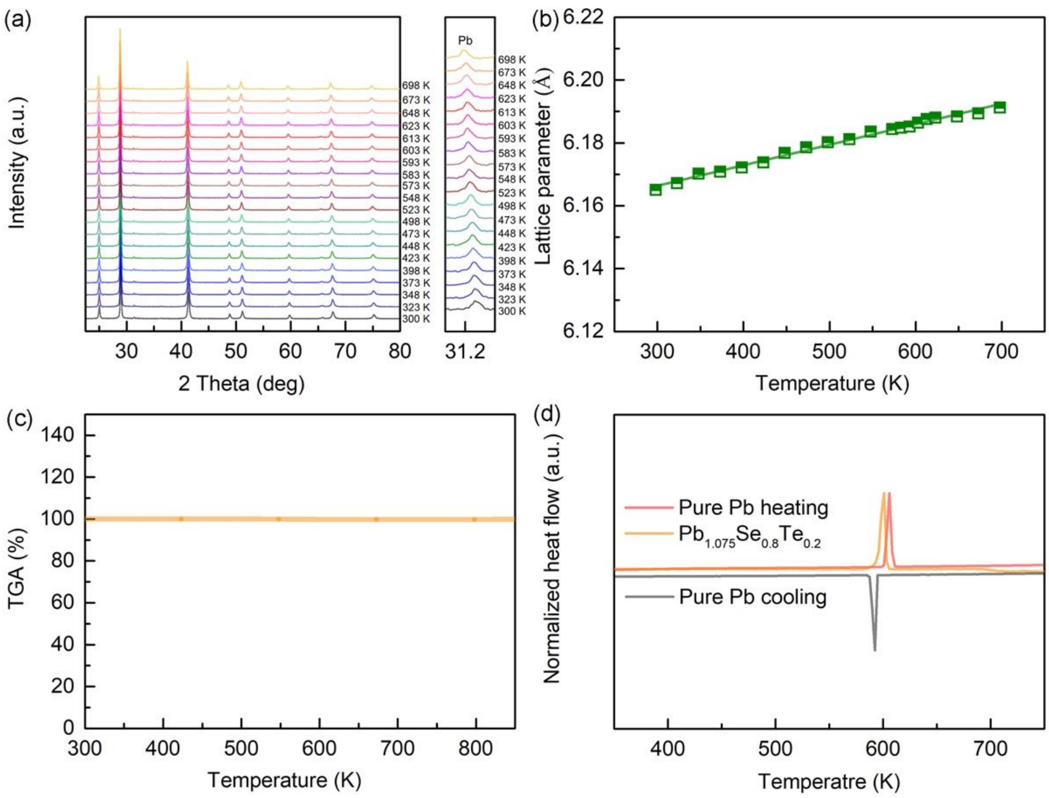

Figure S5. Thermal behavior of the $Pb_{1.075}Se_{0.8}Te_{0.2}$ sample. (a) The in-situ temperature-dependent PXRD patterns. The characteristic Bragg peak of elemental Pb around 31° is magnified in the right side of the panel. (b) The refined lattice parameters with respect to temperature. (c) Thermogravimetric analysis under an Ar flow showing its thermal stability. (d) Differential scanning calorimetry curves for the $Pb_{1.075}Se_{0.8}Te_{0.2}$ sample and pure Pb reference upon the consecutive heating and cooling cycles.

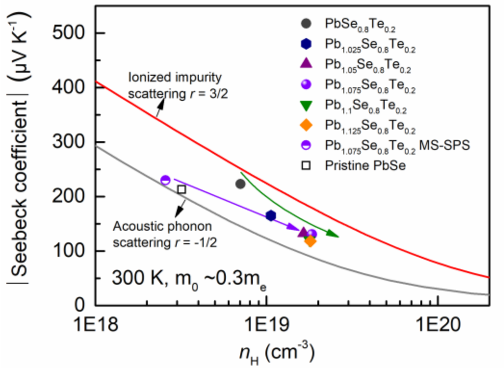

Figure S6. Theoretical Pisarenko relation between the magnitude of S ($|S|$) and n_H calculated based on single parabolic band (SPB) model at 300 K. The theoretical Pisarenko line with the acoustic phonon scattering (r = –1/2) mechanism and density of states effective mass (m_0) of 0.30 m_e is presented as the gray line. The $|S|$ of pristine PbSe[1] and the control sample $Pb_{1.075}Se_{0.8}Te_{0.2}$ prepared by traditional melt-synthesis followed by SPS process (purple circle) are closely located at this line. In contrast, the experimental $|S|$ values of the title $Pb_{1+x}Se_{0.8}Te_{0.2}$ (x = 0 − 0.125) samples lie far above this line and move towards red line (r = 3/2) representing ionized impurity scattering model.

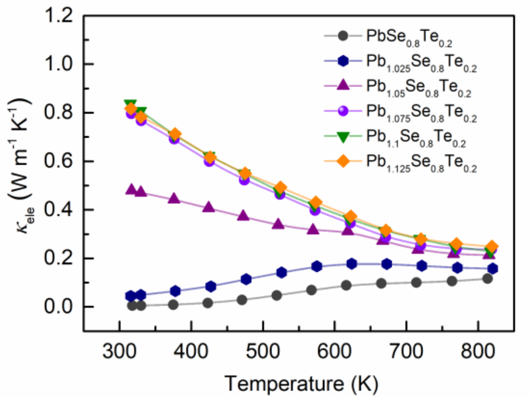

Figure S7. The electronic thermal conductivity (κ_{ele}) of the $Pb_{1+x}Se_{0.8}Te_{0.2}$ samples (x = 0 – 0.125) with respect to temperature.

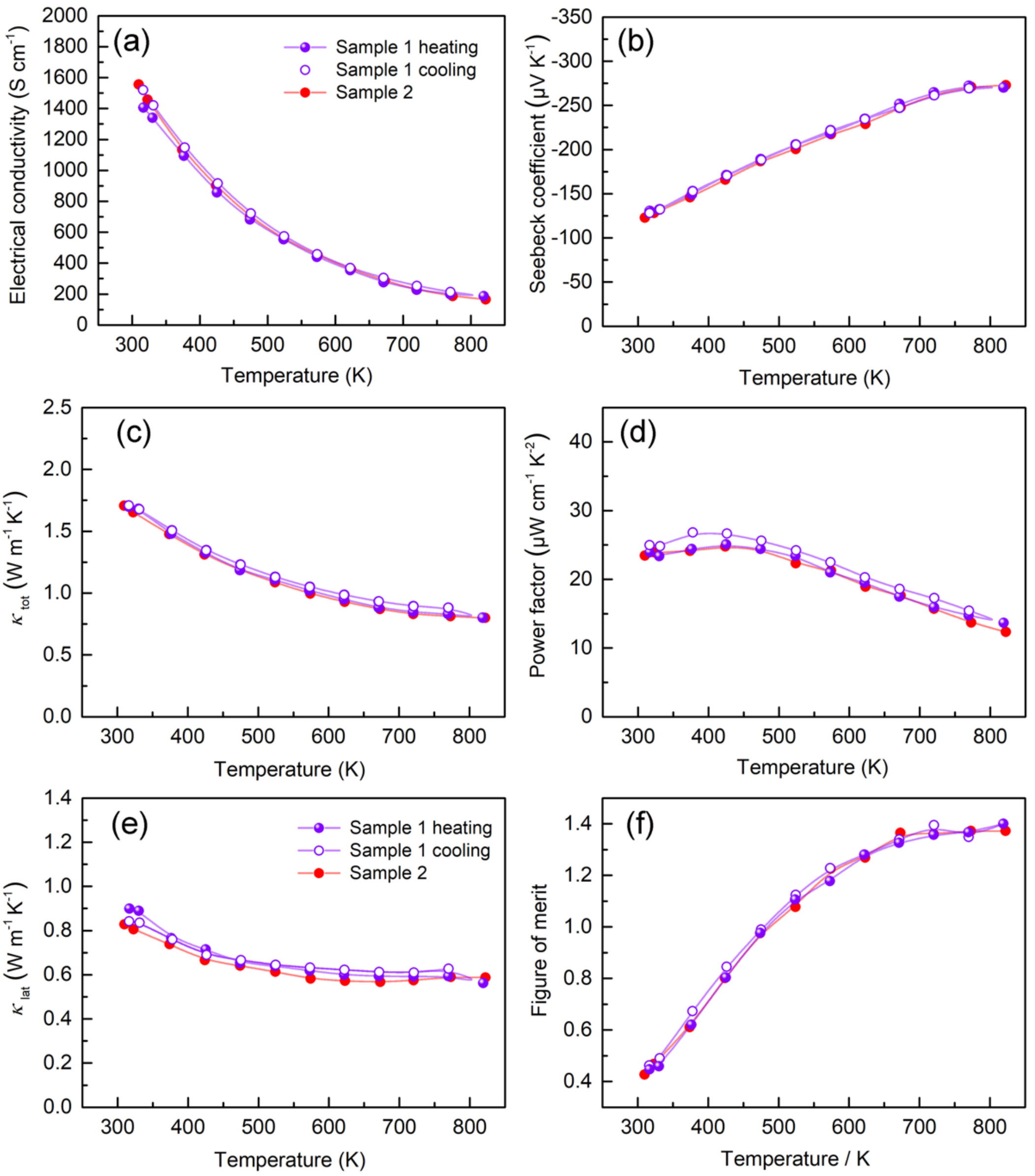

Figure S8. The (a) electrical conductivity (σ), (b) Seebeck coefficient (S), (c) thermal conductivity (κ), (d) power factor (PF), (e) lattice thermal conductivity (κ_{lat}), and (e) thermoelectric figure of merit (ZT) of the $Pb_{1.075}Se_{0.8}Te_{0.2}$ sample. The sample 1 was measured during the consecutive heating and cooling cycle in Seoul National University of Republic of Korea, demonstrating cyclability and thermal stability of the material for thermoelectric power generation in a wide range of temperature. The independently synthesized sample 2 was characterized in Tsinghua University, China, confirming the reproducibility and reliability of thermoelectric performance of the $Pb_{1.075}Se_{0.8}Te_{0.2}$ sample.

4. References

[1] Y. Lee, S. H. Lo, C. Chen, H. Sun, D. Y. Chung, T. C. Chasapis, C. Uher, V. P. Dravid, M. G. Kanatzidis, *Nat. Commun.* **2014**, 5, 3640.

[2] a) L.-D. Zhao, G. Tan, S. Hao, J. He, Y. Pei, H. Chi, H. Wang, S. Gong, H. Xu, V. P. Dravid, C. Uher, G. J. Snyder, C. Wolverton, M. G. Kanatzidis, *Science* **2015**, 351, 141; b) G. Tan, S. Hao, J. Zhao, C. Wolverton, M. G. Kanatzidis, *J. Am. Chem. Soc.* **2017**, 139, 6467.

[3] J. J. Kuo, S. D. Kang, K. Imasato, H. Tamaki, S. Ohno, T. Kanno, G. J. Snyder, *Energy Environ. Sci.* **2018**, 11, 429.

[4] a) C. Zhou, Y. K. Lee, J. Cha, B. Yoo, S. P. Cho, T. Hyeon, I. Chung, *J. Am. Chem. Soc.* **2018**, 140, 9282; b) J. Callaway, H. C. Von Baeyer, *Phys. Rev.* **1960**, 120, 1149.

[5] C. Zhou, Y. Yu, Y. L. Lee, B. Ge, W. Lu, O. Cojocaru-Miredin, J. Im, S. P. Cho, M. Wuttig, Z. Shi, I. Chung, *J. Am. Chem. Soc.* **2020**, 142, 15172.

[6] H. Peng, J.-H. Song, M. G. Kanatzidis, A. J. Freeman, *Phys. Rev. B* **2011**, 84, 125207.

[7] a) Z. Xiao, Y. Zhou, H. Hosono, T. Kamiya, *Phys. Chem. Chem. Phys.* **2015**, 17, 18900; b) S.-H. Wei, *Comput. Mater. Sci.* **2004**, 30, 337.
